# Supplementary material for: Dosage sensitivity to Pumilio1 variants in the mouse brain reflects distinct molecular mechanisms
Source: EMBO J. 2023 Apr 18;42(11):e112721. doi: 10.15252/embj.2022112721 (PMC10233381; doi:10.15252/embj.2022112721)
Supplement: Supplementary file 1 — Appendix S1 [file EMBJ-42-e112721-s009.pdf]

Appendix

Supplemental Figures for:

Dosage sensitivity in Pumilio1 variants reflects distinct molecular mechanisms

Salvatore Botta, Nicola de Prisco, Alexei Chemiakine, Vicky Brandt, Maximilian Cabaj, Purvi Patel, Ella Doron-Mandel, Colton J. Treadway, Marko Jovanovic, Nicholas G. Brown, Rajesh K. Soni, and Vincenzo A. Gennarino

Table of content

Appendix Figure S1-S9

Appendix Fig S1

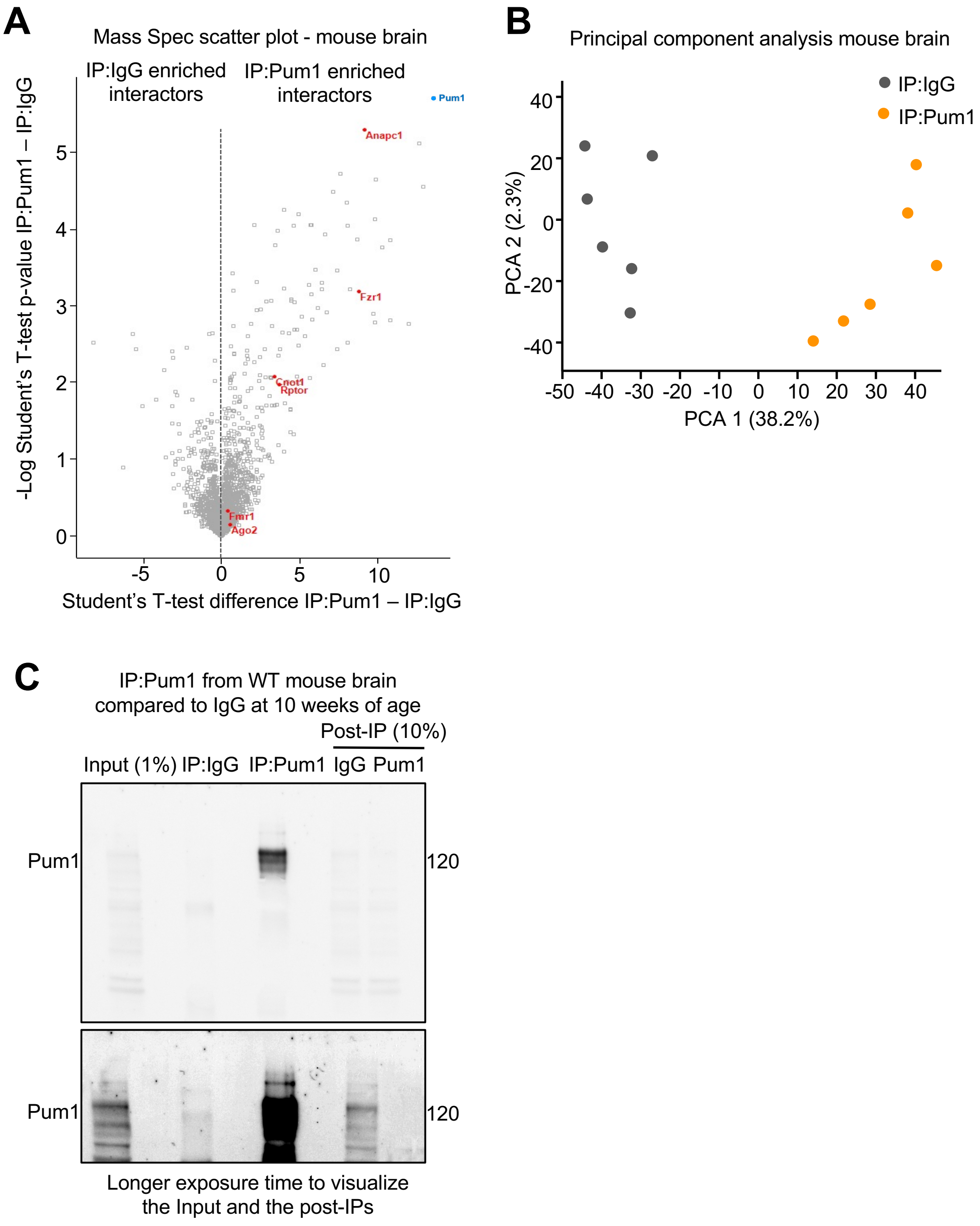

Appendix Figure S1. Pumilio1 antibody efficiency for IP LC-MS/MS. (**A**) Volcano plot analysis showing all the proteins pulled down by IP against IgG and Pum1 from mouse brain. (**B**) Principal component analysis (PCA) of IP-Pum1 followed by LC-MS/MS in WT adult mouse brains compared with IP against IgG; each dot represents one sample for a total of 12 samples processed by LC-MS/MS. (**C**) Pre-IP, IP, and post-IP against Pum1 and IgG from wild-type (WT) mouse brain. Even at very long exposure, the post-IP (10%) Pum1 lane has no residual band at 120 kDa even though 10 times more protein is loaded than Input (1%). This demonstrates the specificity of the Pum1 antibody, which makes it suitable for IP LC-MS/MS. The numbers on the right show molecular weight in kilodaltons (kDa).

Appendix Fig S2

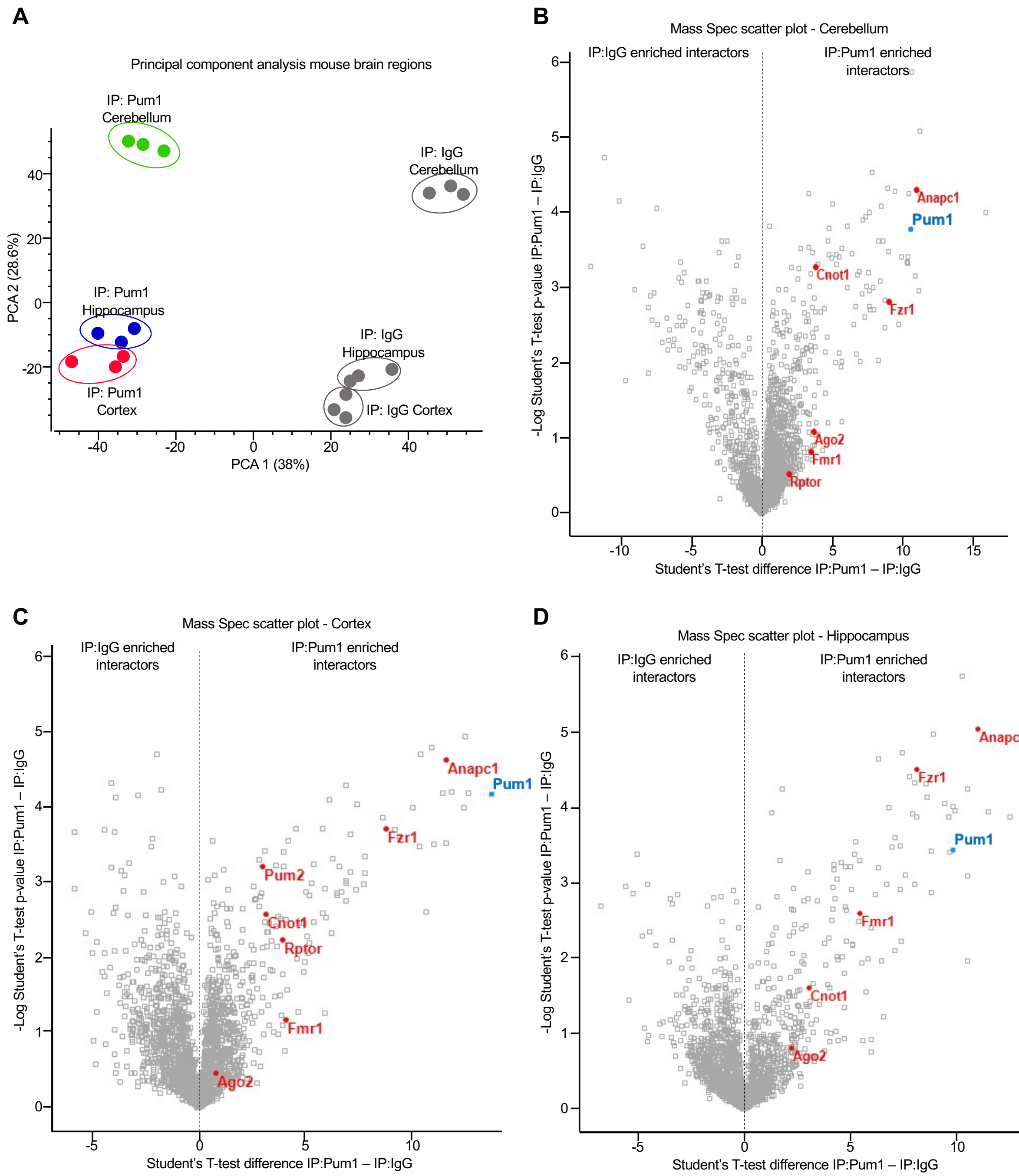

Appendix Figure S2. Volcano plot and PCA analyses of IP:Pum1 followed by LC-MS/MS in cerebellum, hippocampus, and cortex. (A) Principal component analysis (PCA) of IP-Pum1 followed by LC-MS/MS in cortex, hippocampus, and cerebellum from WT mice. (**B-D**) Volcano plots show all the proteins pulled down by IP against IgG and Pum1 from (**B**) cerebellum, (**C**) cortex, and (**D**) hippocampus at 10 weeks of age. IP against IgG was used as a negative control. Each dot represents a total of 3 samples processed by MS for each brain region. All putative Pum1 interactors are listed in **Table EV1**.

Appendix Fig S3

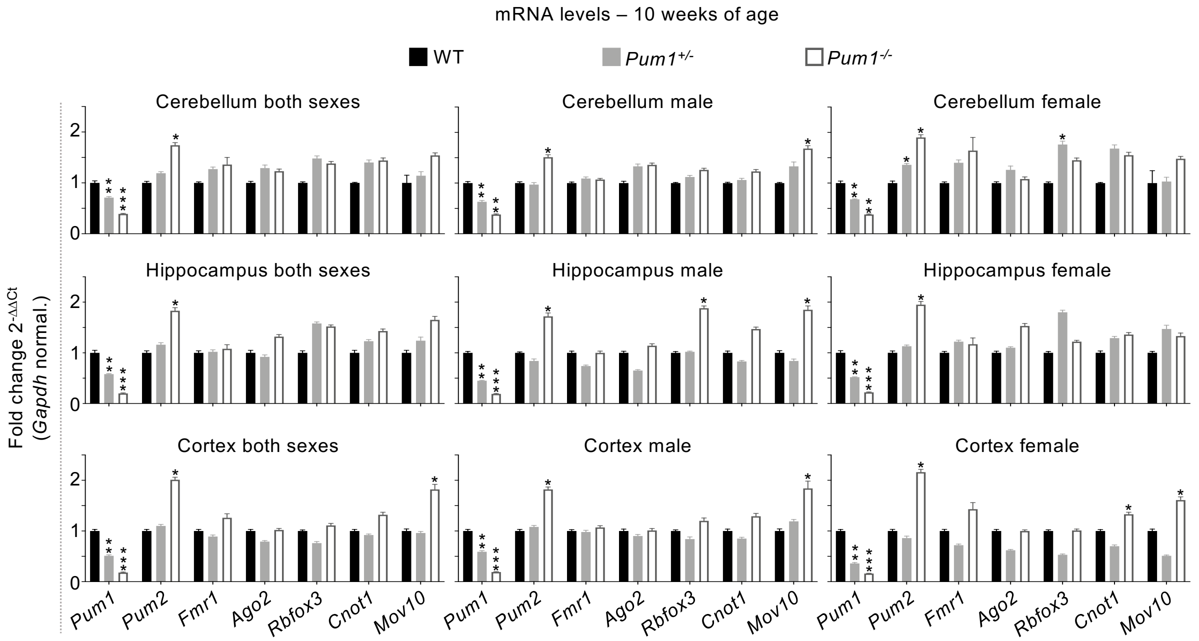

Appendix Figure S3. mRNA quantification of Pum1 interactors by brain region and sex in WT, *Pum1*$^{+/-}$ and *Pum1*$^{-/-}$ mice. mRNA levels in cerebellum, hippocampus, and cortex in male and female for all the validated Pum1 interactors. The same number of mice were used here as in **Fig 4A-C** for a total of at least 12 mice per genotype and sex at 10 weeks of age. All data were normalized to *Gapdh* mRNA levels. All the experiments were performed at least six times (data represent mean ± SEM). The *p* values were calculated by the Student's t test. *$p < 0.05$, **$p < 0.01$, ***$p < 0.001$.

Appendix Fig S4

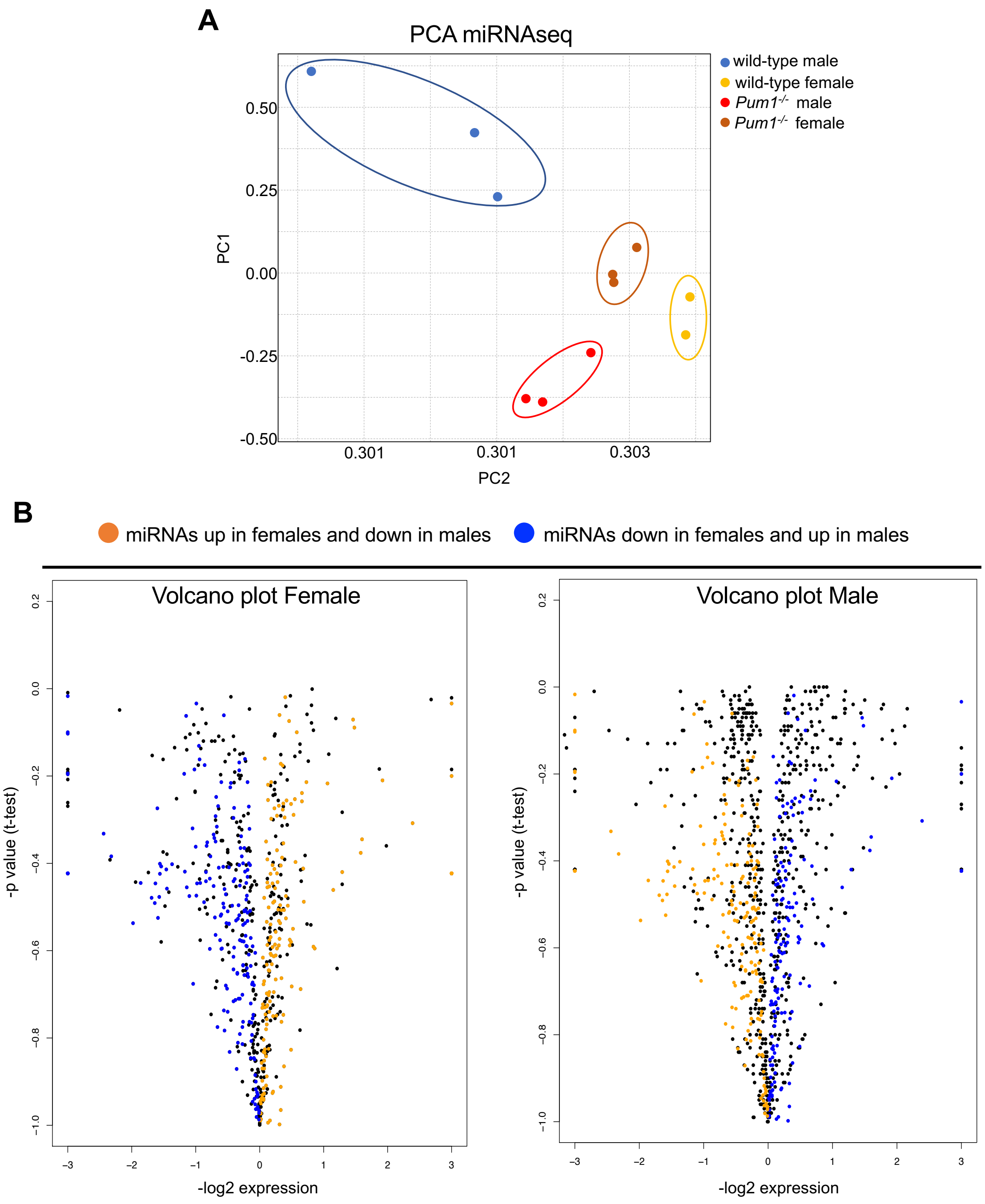

Appendix Figure S4. Volcano plots representing all the miRNAs sequenced by miRNAseq in male and female. (A) Principal component analysis (PCA) of miRNAseq in wild-type and *Pum1*$^{-/-}$ male and female mice cerebella at 10 weeks of age. (**B**) Volcano plots show the expression profile for all the miRNAs in male and female *Pum1*$^{-/-}$ mice compared to WT at 10 weeks of age. The orange dots represent the miRNAs upregulated in female and downregulated in males; the blue dots represent the miRNAs downregulated in female and upregulated in males (see Material and Methods).

Appendix Fig S5

RNA quantification from male and female cerebellum at 10 weeks of age.
Genes plotted here sorted by number of co-bound miRNAs from **Figure 4D** with at least 25% expression change

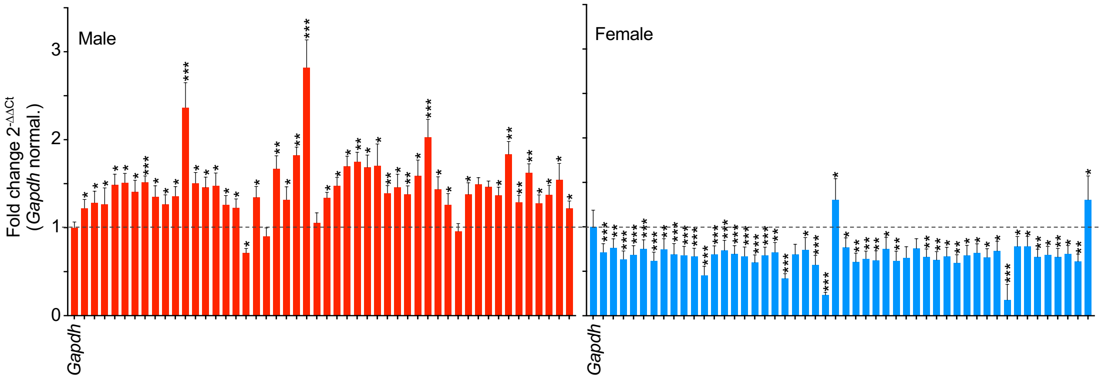

Table showing the order of gene represented in the figure above starting from *Gapdh* as represented in Figure 4E

Position in the graph	Gene	Male	Female	Error_Male	Error_Female	Co-bound miRNAs
1	*Gapdh*	1.000	1.000	0.065	0.188	11
2	*Aak1*	1.222	0.714	0.099	0.096	11
3	*Plekhm3*	1.283	0.764	0.132	0.102	11
4	*Zbtb20*	1.268	0.636	0.185	0.093	10
5	*Ago3*	1.485	0.686	0.122	0.102	10
6	*Dgkh*	1.511	0.751	0.106	0.103	10
7	*Fto*	1.407	0.618	0.132	0.097	10
8	*Grin2b*	1.517	0.749	0.071	0.117	10
9	*Lpp*	1.353	0.689	0.124	0.123	10
10	*Stx17*	1.266	0.682	0.108	0.098	10
11	*Acvr2b*	1.359	0.668	0.108	0.092	9
12	*Ap5m1*	2.366	0.456	0.282	0.101	9
13	*Chl1*	1.506	0.691	0.121	0.094	9
14	*Gpr161*	1.457	0.735	0.118	0.113	9
15	*Kcnn3*	1.477	0.694	0.145	0.094	9
16	*Klf7*	1.262	0.670	0.103	0.103	9
17	*Nfat5*	1.228	0.598	0.099	0.086	9
18	*Plxna4*	0.712	0.680	0.051	0.092	9
19	*Slc1a2*	1.344	0.713	0.125	0.111	9
20	*Slc8a1*	0.904	0.417	0.094	0.056	9
21	*Ston2*	1.669	0.690	0.148	0.114	9
22	*Tsc22d2*	1.319	0.743	0.146	0.137	9
23	*Xkr4*	1.823	0.573	0.093	0.105	9
24	*A1cf*	2.821	0.232	0.314	0.031	8
25	*Aff4*	1.053	1.304	0.116	0.238	8
26	*Clstn2*	1.340	0.768	0.062	0.109	8
27	*Cnnm2*	1.475	0.605	0.097	0.099	8
28	*Csnk1a1*	1.698	0.641	0.115	0.087	8
29	*Ctdspl2*	1.749	0.624	0.108	0.108	8
30	*Dcaf7*	1.685	0.750	0.141	0.132	8
31	*Fmnl3*	1.705	0.620	0.246	0.106	8
32	*Frmd4a*	1.394	0.654	0.085	0.123	8
33	*Grin2a*	1.459	0.755	0.148	0.112	8
34	*Hipk2*	1.381	0.662	0.094	0.088	8
35	*Kif26b*	1.588	0.627	0.181	0.094	8
36	*Klf12*	2.030	0.668	0.202	0.101	8
37	*Lmln*	1.435	0.595	0.143	0.091	8
38	*Lrrc40*	1.262	0.679	0.126	0.113	8
39	*Myo5a*	0.958	0.707	0.089	0.102	8
40	*Nav2*	1.377	0.658	0.135	0.097	8
41	*Psd3*	1.493	0.728	0.076	0.110	8
42	*Ptbp2*	1.463	0.177	0.068	0.177	8
43	*Rimkla*	1.368	0.779	0.092	0.112	8
44	*Snx30*	1.833	0.781	0.147	0.112	8
45	*Taok1*	1.290	0.660	0.078	0.088	8
46	*Zbtb10*	1.624	0.686	0.102	0.092	8
47	*Apbb2*	1.278	0.660	0.094	0.097	8
48	*Acap2*	1.374	0.697	0.107	0.093	8
49	*Acer2*	1.544	0.610	0.187	0.083	8
50	*Aebp2*	1.219	1.904	0.085	0.269	8

Appendix Figure S5. mRNA quantification of the 49 targets co-bound by at least eight dysregulated miRNAs in mouse cerebellum. qPCR in cerebellum of male (*left*, red) and female (*right*, blue) mice at 10 weeks of age for the 49 targets co-bound by at least eight dysregulated miRNAs (with minimum 25% change in expression) from **Fig 4E** and **Table EV4**. All the experiments were performed in triplicate for both male and female (data represent mean ± SEM). The p values were calculated by two-tailed Student's t test. *$p < 0.05$, **$p < 0.01$, ***$p < 0.001$.

Appendix Fig S6

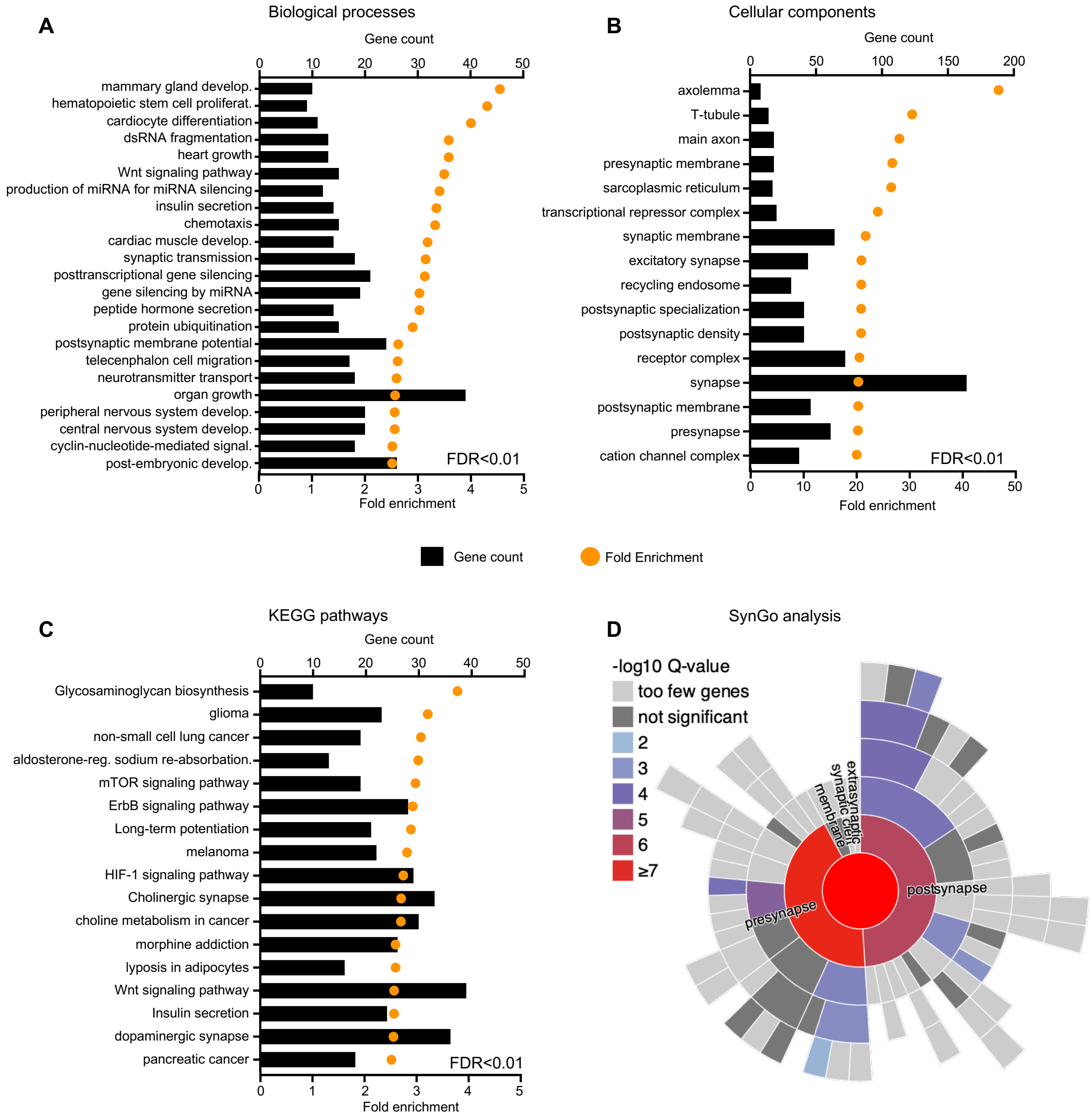

Appendix Figure S6. Gene ontology analysis for all targets predicted by CoMeTa and TargetScan that are co-bound by at least four miRNAs. (**A-C**) David Gene Ontology representing the enriched (**A**) biological processes, (**B**) cellular components, and (**C**) KEGG pathways for all the targets co-bound by at least four miRNAs. For this analysis we set FDR<0.01 and a fold-enrichment >2. (**D**) Synaptic Gene Ontology (SynGO) predicts that 117 targets are presynaptic and 124 are postsynaptic with a log10Q value≥5.

Appendix Fig S7

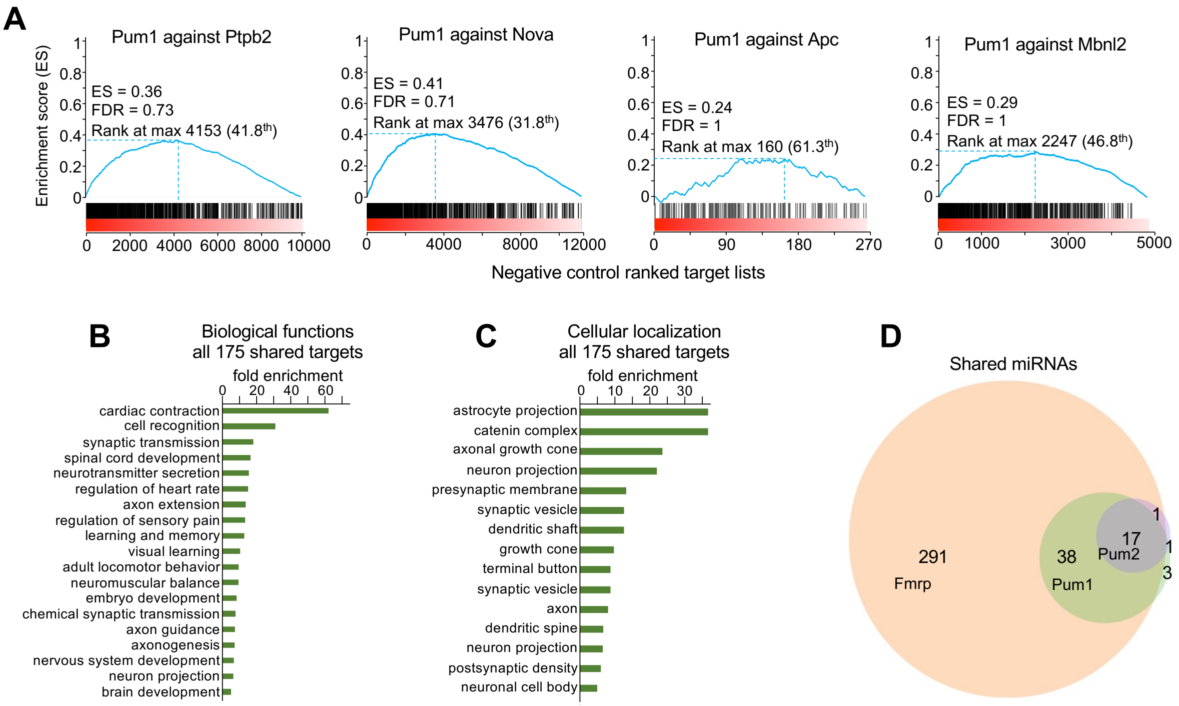

Appendix Figure S7. GSEA and Gene Ontology data pertaining to Figure 5. (**A**) Gene Set Enrichment Analysis (GSEA) of Pum1 HITS-CLIP data plotted against HITS-CLIP data from the negative controls (RBPs that did not show up in the Pum1 interactome: Ptpb2, Nova, Apc, and Mbnl2) reveals no significant enrichment. (**B**-**C**) Gene ontology analysis of the HITS-CLIP targets shared between Pum1, Pum2, Fmrp, Ago2, and Rbfox3 reveals enrichment for certain (**B**) biological functions and (**C**) cellular localization. Only categories with FDR<0.05 and fold enrichment > 5 were plotted in **B** and **C**. (**D**) Venn diagram of miRNAs identified by Pum1 and Pum2 shows almost 100% overlap with the miRNAs pulled down by Fmrp HITS-CLIP. For full list of shared miRNAs see **Table EV6**. For all GSEA analyses the False Discovery Rate (FDR) was provided by GSEA, ***FDR < 0.01. ES=Enrichment score (blue line).

Appendix Fig S8

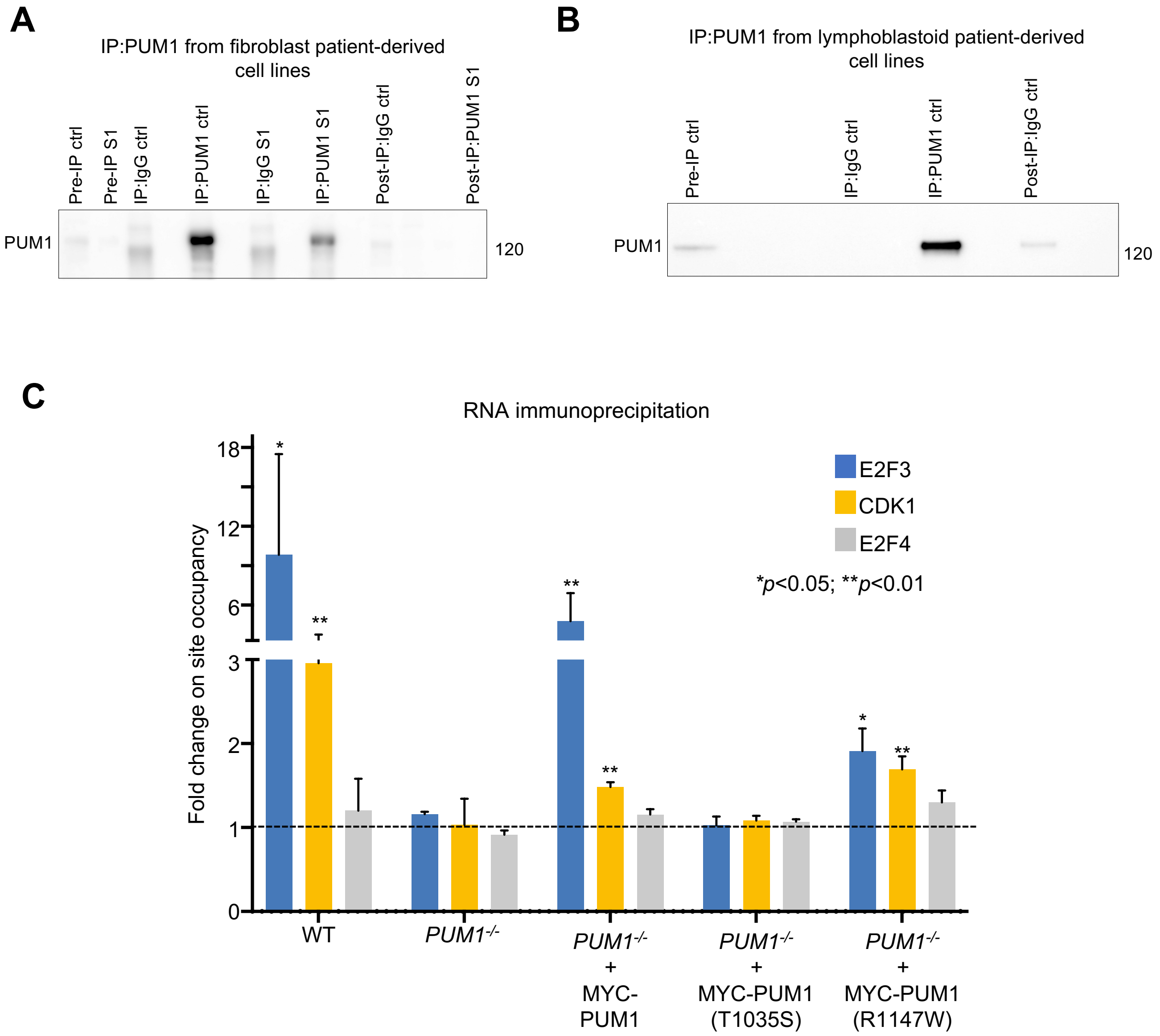

Appendix Figure S8. PUM1 R1147W retains RNA-binding activity. (**A-B**) Pre-IP, IP, and post-IP against PUM1 and IgG from (**A**) R1147W (infantile-onset SCA7, or PADDAS) fibroblasts and (**B**) T1035S (adult-onset SCA47 or PRCA) lymphoblastoid cells. In both cell lines we were able to pull down 100% of PUM1. Pre-IP represents 1% from the initial protein lysate as a loading control, while 10% of the protein lysate was loaded as post-IP. Molecular weights provided at right in kilodaltons (kDa). **(C)** RNA-immunoprecipitation assay followed by qRT-PCR to show that T1035S is impaired in RNA-binding while R1147W is not. We used *E2F3* and *CDK1* as validated PUM1 targets and E2F4 as a negative control. We transfected WT and PUM1-KO HCT116 cells with 250ng of either Myc-PUM1-WT, Myc-PUM1-R1147W, or Myc-PUM1-T105S. RIP was performed in triplicate.

Appendix Fig S9

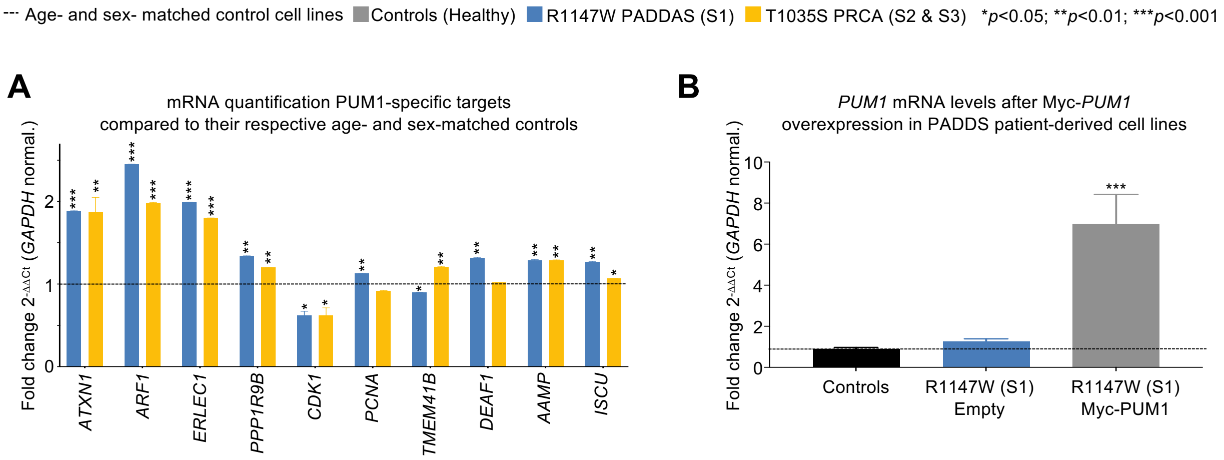

Appendix Figure S9. mRNA quantification of PUM1-specific targets. (**A**) qPCR analysis of validated PUM1-specific targets from PADDAS (R1147W) patient-derived fibroblasts (blue bars) compared to three age- and sex-matched fibroblast control cell lines, and PRCA (T1035S) patient-derived lymphoblastoid cell lines (orange bars) compared to three age- and sex-matched lymphoblastoid control cell lines. Only genes expressed in both fibroblasts and lymphoblasts are represented here, for a total of 10 genes. (**B**) mRNA quantification of PUM1 from PADDAS patient-derived fibroblasts transfected with empty and Myc-PUM1-WT vectors, compared to three age- and sex-matched fibroblast control cell lines. All data were normalized to *GAPDH* mRNA levels and experiments performed at least three times. Data represent mean ± SEM. P values were calculated by two-tailed Student's t test. *$p < 0.05$, **$p < 0.01$, ***$p < 0.001$.
