# Supplementary material for: Dosage sensitivity to Pumilio1 variants in the mouse brain reflects distinct molecular mechanisms
Source: EMBO J. 2023 Apr 18;42(11):e112721. doi: 10.15252/embj.2022112721 (PMC10233381; doi:10.15252/embj.2022112721)
Supplement: Supplementary file 2 — Expanded View Figures PDF [file EMBJ-42-e112721-s012.pdf]

Expanded View Figures

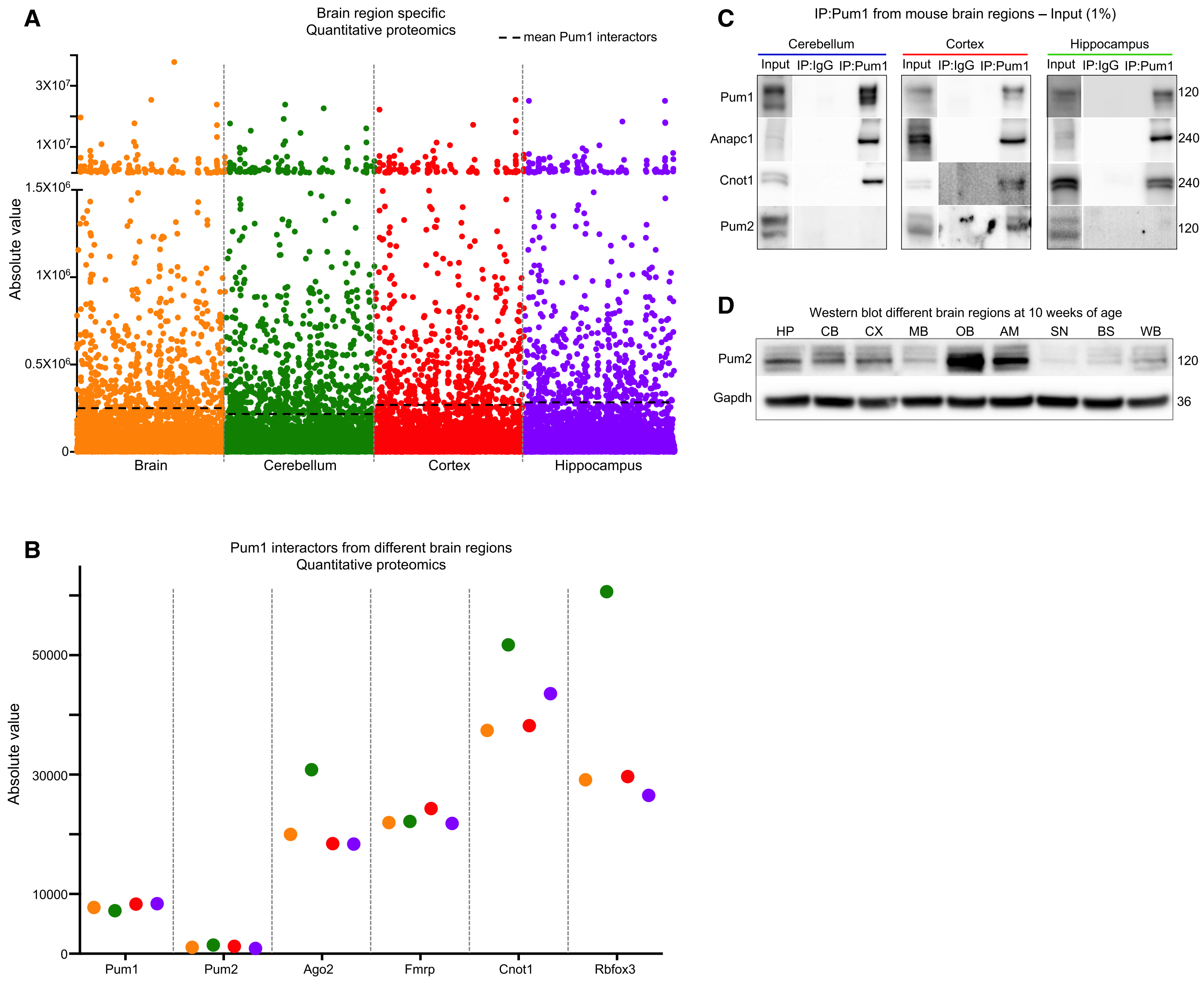

Figure EV1. Many Pum1 interactors are specific to certain brain regions and not determined by expression level.

A Proteomic analysis of the whole brain, cerebellum, hippocampus, and cortex at 10 weeks of age shows that Pum1 interactors are not the most highly expressed proteins. Dotted line represents the mean expression of Pum1 interactors from each brain region. Proteomics was performed in duplicate (one male and one female) for each brain region.

B Absolute expression value of the validated Pum1 interactors. Pum2 is expressed at low levels in all three brain regions but was still the strongest Pum1 interactor in the cortex, suggesting a specific interaction rather than a post-lysis artifact.

C Immunoblot for Pum1 (positive control), Anapc1, Cnot1 and Pum2.

D Western blot analysis at 10 weeks of age to evaluate Pum2 expression levels in eight different brain regions as well as whole brain. Pum2 is highly expressed in the olfactory bulbs and amygdala, and expressed at similar levels in the hippocampus, cerebellum, and cortex.

Data information: Cerebellar and cortical tissues: $n = 8$ wild-type mice (four male and four female), for a total of 24 mice. Hippocampus: $n = 10$ wild-type mice (five female and five male), for a total of 30 mice. All mice were 10 weeks of age. IP against IgG was used as a negative control. Molecular protein weights are expressed in kilodaltons (kDa). AM, amygdala; BS, brain stem; CB, cerebellum; CX, cortex; HP, hippocampus; MB, midbrain; OB, olfactory bulbs; SN, substantia nigra pars compacta; WB, whole brain. All the experiments were repeated at least three times.

 © 2023 The Authors

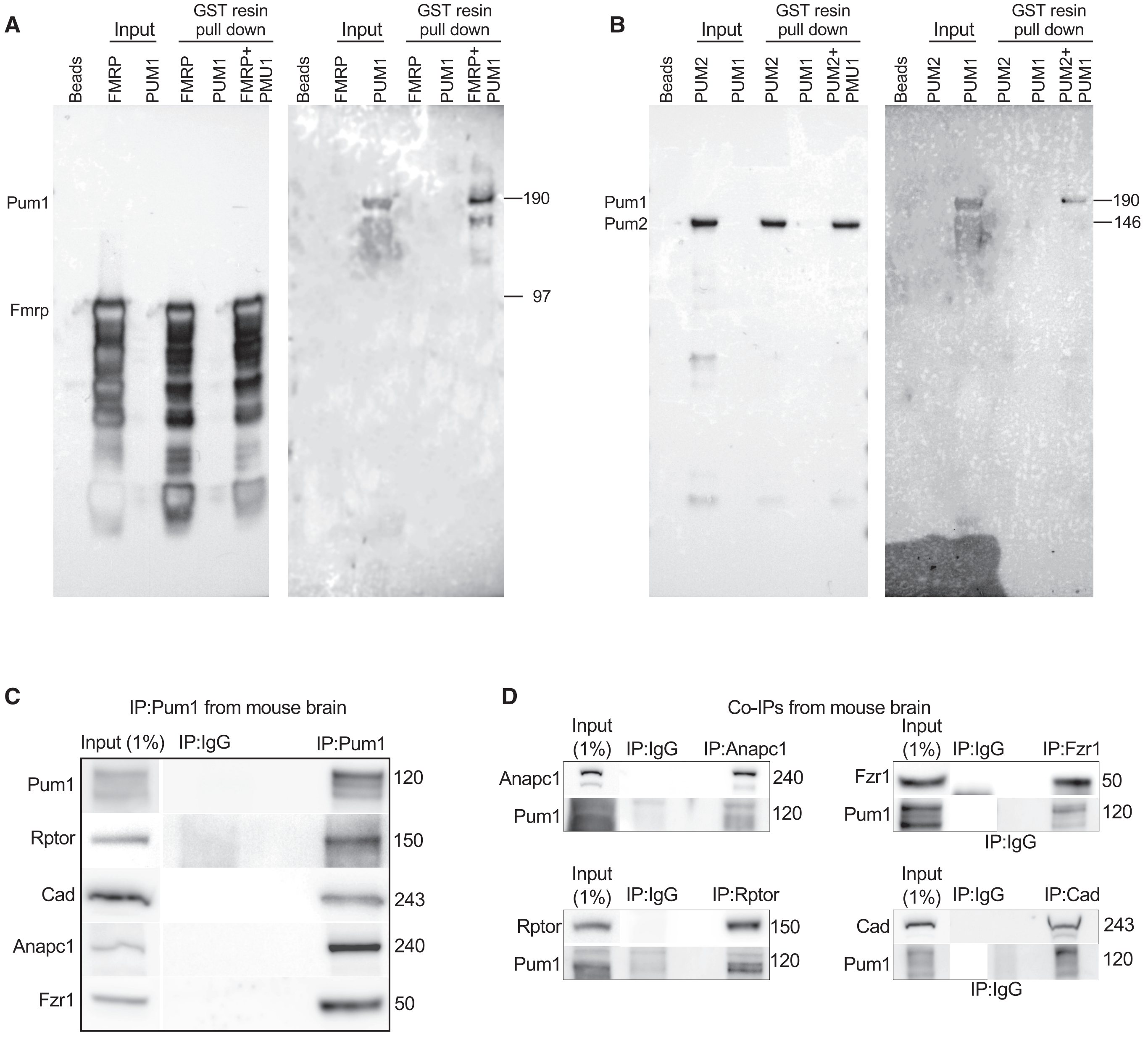

Figure EV2. Creation of recombinant, GST-tagged Fmrp and Pum2 and validation of APC/C and mTOR association with PUM1.

A, B Representative western blot of PUM1 pulled down by recombinant (A) GST-FMRP or (B) GST-PUM2.

C Representative western blot of Rptor, Cad, Anapc1 and Fzr1 proteins pulled down by IP against Pum1 (used here as positive control) from WT mice.

D Representative western blot of the reciprocal co-IPs against Anapc1, Fzr1, Rptor, and Cad. Each co-IP was immunoblotted against Pum1 and the pulled down protein used here as a reference protein to confirm the respective protein–protein interaction.

Data information: For (C) and (D), IP against IgG was used as a negative control, and Input (1% from the initial protein lysate) as a loading control. Molecular weights are expressed in kilodaltons (kDa) to the right. All mice were sacrificed at 10 weeks of age with an equal number or male and female.

© 2023 The Authors

Figure EV3. Pum1 antibody specificity and evaluation of RBP associations with Pum1 in mouse and HEK293T cells, with and without RNase.

A IP against Pum1 in *Pum1*$^{-/-}$ mouse demonstrates the complete absence of Pum1 and thus the specificity of the anti-Pum1 antibody. IP against IgG was used as a negative control, and Input (1% from the initial protein lysate) as a loading control.

B IP against Pum1 (with or without RNase treatment) shows no interaction with Ago1 or Ago3 in the mouse brain.

C Representative western blots of the same proteins validated in Fig 3 after IP against PUM1 with or without RNase treatment from HEK293T cell lines. IP against IgG was used as a negative control and Input (1% from the initial protein lysate) as a loading control.

Data information: The numbers on the right are the respective molecular weights expressed in kilodaltons (kDa). All mice were sacrificed at 10 weeks of age.

© 2023 The Authors

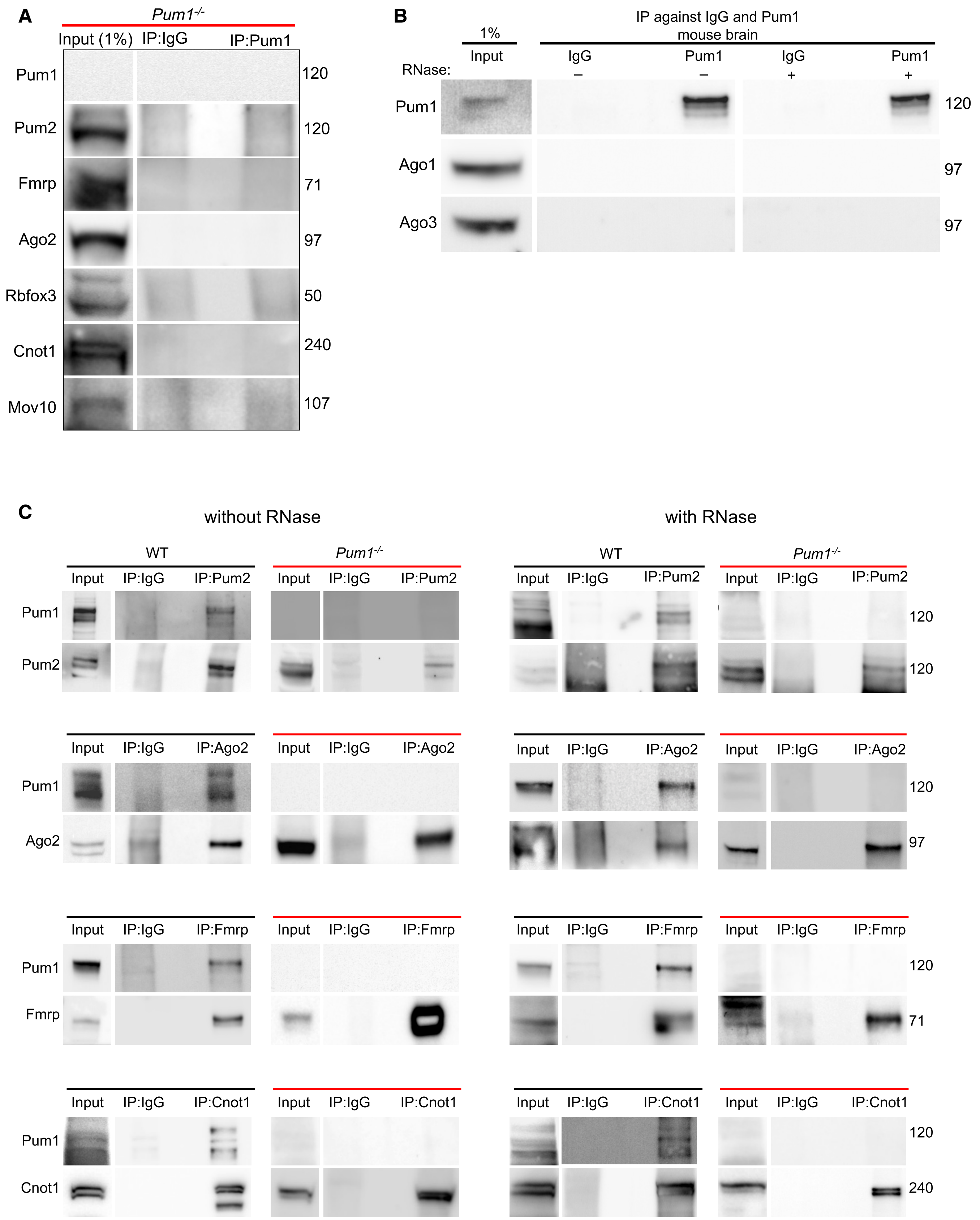
A
Pum1-/-
Input (1%)
IP:IgG
IP:Pum1
Pum1
Pum2
Fmrp
Ago2
Rbfox3
Cnot1
Mov10
120
120
71
97
50
240
107
B
IP against IgG and Pum1
mouse brain
1%
Input
IgG
Pum1
IgG
Pum1
RNase:
–
–
+
+
Pum1
Ago1
Ago3
120
97
97
C
without RNase
with RNase
WT
Pum1-/-
Input
IP:IgG
IP:Pum2
IP:Ago2
IP:Fmrp
IP:Cnot1
Pum1
Pum2
Ago2
Fmrp
Cnot1
120
120
120
97
120
71
120
240

Figure EV3.

Figure EV4. Protein and mRNA quantification from WT, *Pum1*$^{+/-}$ and *Pum1*$^{-/-}$ mouse brains.

A Representative western blot with relative quantifications of Pum1, Pum2, Fmrp, Ago2, Rbfox3, Cnot1, and Mov10 from whole brains of WT, *Pum1*$^{+/-}$ and *Pum1*$^{-/-}$ mice. All data were normalized to Gapdh protein levels. The numbers on the right are the respective molecular weights in kilodaltons (kDa).

B mRNA level quantification by qPCR of *Pum1*, *Pum2*, *Fmrp*, *Ago2*, *Rbfox3*, *Cnot1*, and *Mov10* from whole brains of WT, *Pum1*$^{+/-}$ and *Pum1*$^{-/-}$ mice.

Data information: All data were normalized to *Gapdh* mRNA levels. All the experiments were conducted with equal numbers of male (at least 3 per genotype) and female (at least 3 per genotype) mice at 10 weeks of age, for a total of at least 6 mice per genotype (data represent mean ± SEM). The *p* values were calculated by the Student's *t* test. *$P < 0.05$, **$P < 0.01$, ***$P < 0.001$.

© 2023 The Authors

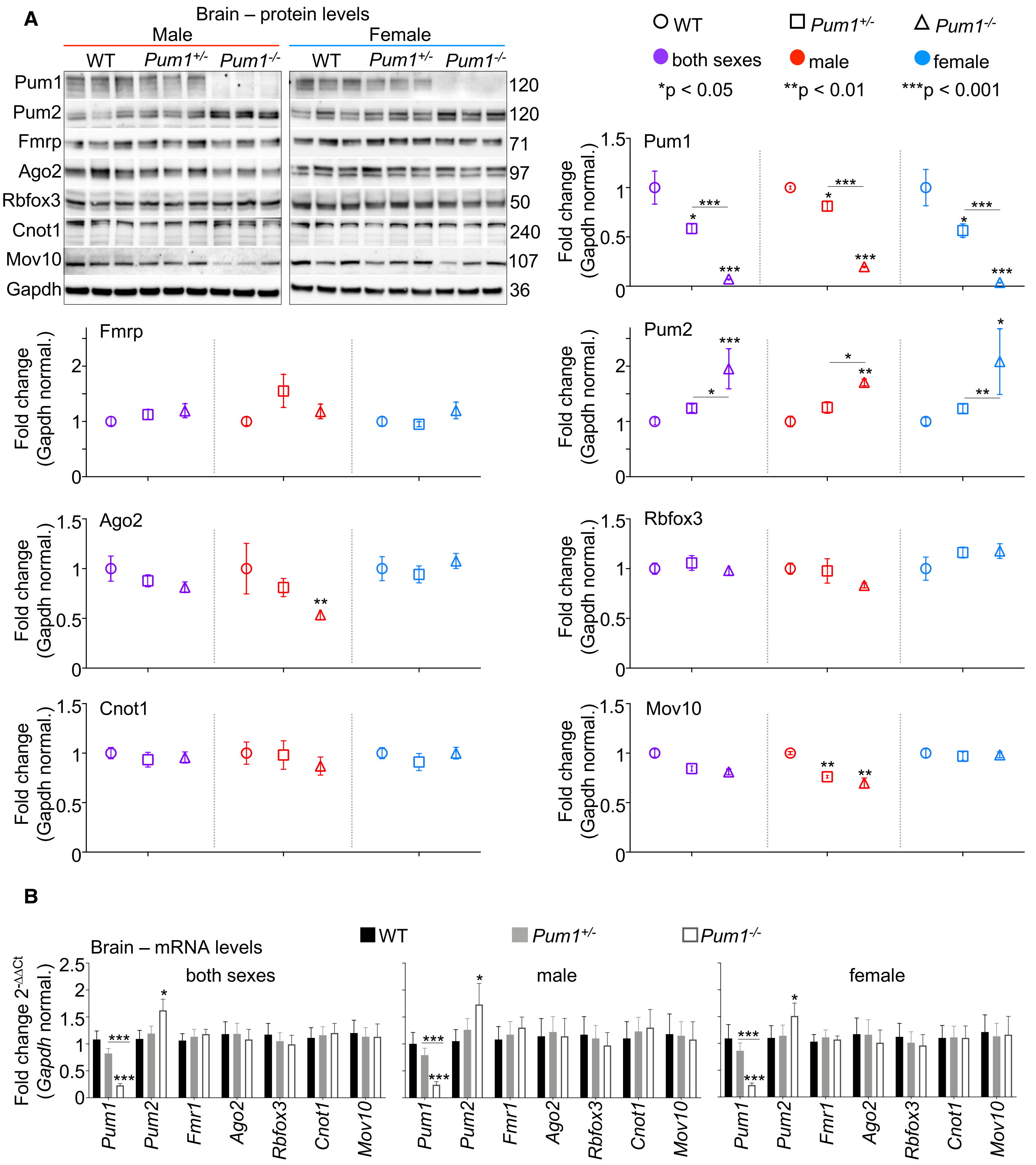
A
Brain – protein levels
Male
Female
WT
Pum1+/-
Pum1-/-
Pum1
Pum2
Fmrp
Ago2
Rbfox3
Cnot1
Mov10
Gapdh
120
120
71
97
50
240
107
36
WT
Pum1+/-
Pum1-/-
both sexes
male
female
*p < 0.05
**p < 0.01
***p < 0.001
Fold change (Gapdh normal.)
B
Brain – mRNA levels
both sexes
male
female
Fold change 2-ΔΔCt (Gapdh normal.)
Pum1
Pum2
Fmr1
Ago2
Rbfox3
Cnot1
Mov10

Figure EV4.

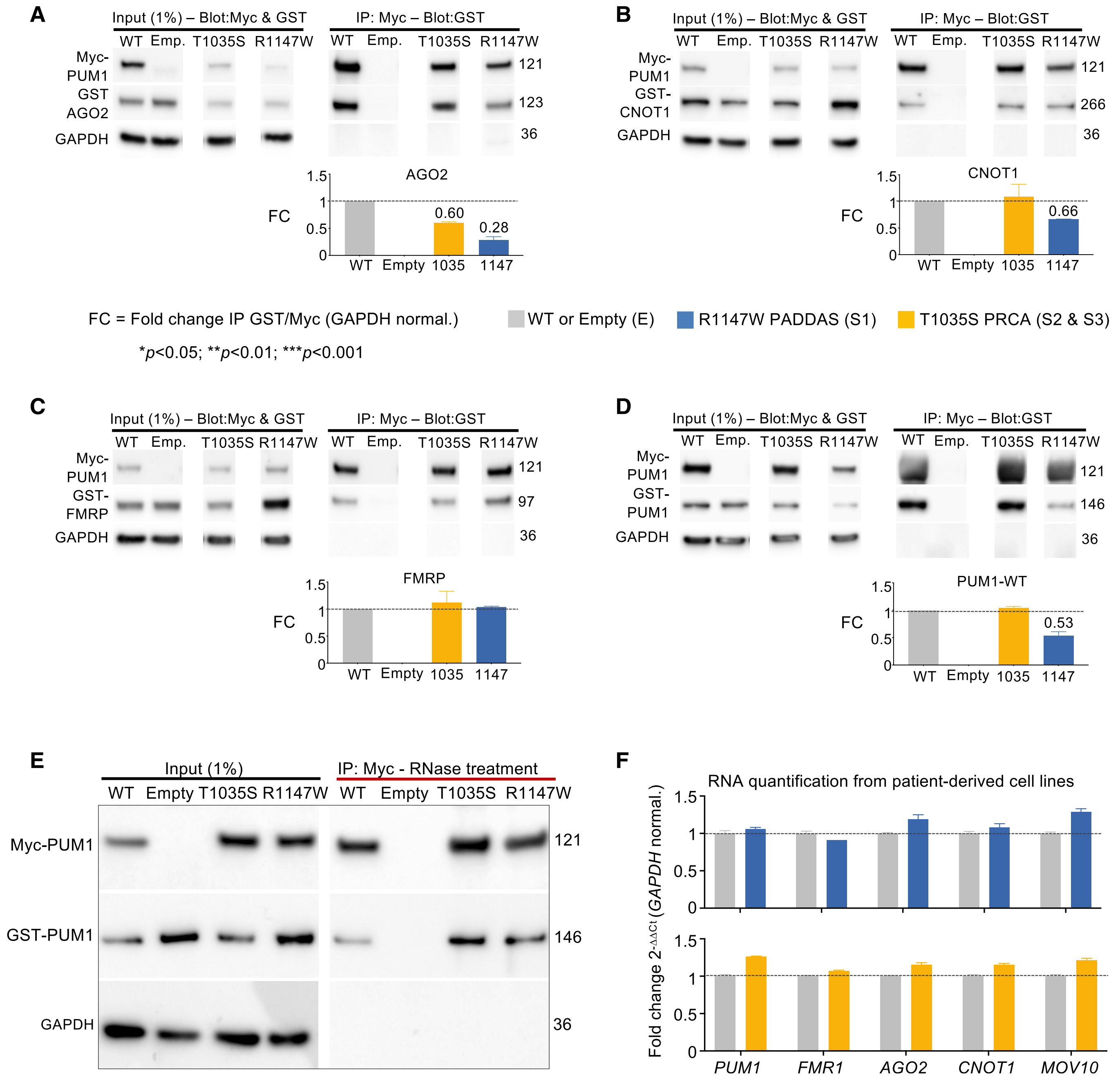

Figure EV5. ***In vitro* validation experiments with tagged proteins.**

A–D Representative western blots and relative IP quantification (*bar graphs*) of IP against Myc-PUM1-WT, Myc-PUM1-T1035S (PRCA), or Myc-PUM1-R1147W (PADDAS) followed by immunoblotting for: (A) GST-AGO2, (B) GST-CNOT1, (C) GST-FMRP, and (D) GST-PUM1-WT. Myc- and GST-tagged proteins were co-transfected in HEK293T cells in equal quantities (250 ng each). The molecular weights to the right are in kilodaltons (kDa). GAPDH was used here as loading control, see Materials and Methods for quantification.

E Representative western blots of IP with RNase treatment against Myc-PUM1-WT, Myc-PUM1-T1035S (PRCA), and Myc-PUM1-R1147W (PADDAS) followed by immunoblotting to test binding between PUM1 proteins without the RNA. The numbers on the right are molecular weights, expressed in kilodaltons (kDa). All the IPs were repeated at least three times.

F mRNA quantification for all of the immunoblotted proteins in Fig 6C in PADDAS and PRCA patient-derived cell lines compared with their respective age-, sex-, and cell-type-matched controls.

Data information: All the IPs and RNA quantification were repeated at least three times. In all the experiments, data represent mean ± SEM. *P* values were calculated by two-tailed Student's *t* test. $^*P < 0.05$, $^{**}P < 0.01$, $^{***}P < 0.001$.

© 2023 The Authors
