# Supplementary material for: Dosage sensitivity to Pumilio1 variants in the mouse brain reflects distinct molecular mechanisms
Source: EMBO J. 2023 Apr 18;42(11):e112721. doi: 10.15252/embj.2022112721 (PMC10233381; doi:10.15252/embj.2022112721)
Supplement: Supplementary file 14 [file EMBJ-42-e112721-s006.pdf]

THE EMBO JOURNAL

Dosage sensitivity to Pumilio1 variants reflects distinct molecular mechanisms

Salvatore Botta, Nicola de Prisco, Alexei Chemiakine, Vicky Brandt, Maximilian Cabaj, Purvi Patel, Ella Doron-Mandel, Colton Treadway, Marko Jovanovic, Nicholas Brown, Rajesh Soni, and Vincenzo Gennarino
DOI: 10.15252/embj.2022112721

Corresponding author(s): Vincenzo Gennarino (vag2138@cumc.columbia.edu)

Review Timeline:

Submission Date:	29th Sep 22
Editorial Decision:	23rd Nov 22
Revision Received:	6th Jan 23
Editorial Decision:	20th Feb 23
Revision Received:	1st Mar 23
Accepted:	14th Mar 23

Editor: Kelly Anderson

Transaction Report:

(Note: With the exception of the correction of typographical or spelling errors that could be a source of ambiguity, letters and reports are not edited. Depending on transfer agreements, referee reports obtained elsewhere may or may not be included in this compilation. Referee reports are anonymous unless the Referee chooses to sign their reports.)

1st Editorial Decision 23rd Nov 2022

Dear Prof. Gennarino,

Thank you for submitting your manuscript for consideration by the EMBO Journal. It has now been seen by three referees whose comments are shown below.

Given the referees' recommendations, I would like to invite you to submit a revised version of the manuscript, addressing the comments of all three reviewers. I should add that it is EMBO Journal policy to allow only a single round of revision, and acceptance of your manuscript will therefore depend on the completeness of your responses in this revised version.

When preparing your letter of response to the referees' comments, please bear in mind that this will form part of the Review Process File, and will therefore be available online to the community. For more details on our Transparent Editorial Process, please visit our website: https://www.embopress.org/page/journal/14602075/authorguide#transparentprocess

We generally allow three months as standard revision time. As a matter of policy, competing manuscripts published during this period will not negatively impact on our assessment of the conceptual advance presented by your study. However, we request that you contact the editor as soon as possible upon publication of any related work, to discuss how to proceed. Should you foresee a problem in meeting this three-month deadline, please let us know in advance and we may be able to grant an extension. I have attached a guide for revisions to this email for your convenience.

Thank you for the opportunity to consider your work for publication. I look forward to your revision.

Yours sincerely,

Kelly

Kelly M Anderson, PhD
Editor
The EMBO Journal
k.anderson@embojournal.org

Referee #1:

In the present manuscript "Dosage sensitivity in Pumilio1-related phenotypes reflects distinct disease mechanisms" by Botta and colleagues aimed at unraveling why differences in expression of the RNA binding protein (RBP) Pumilio1 (Pum1) caused by pathogenic mutations results in clearly different phenotypes. To this end, the authors analyzed the interactome of Pum1 in different brain regions. In addition, the authors investigated how reduced levels of Pum1 (due to pathogenic mutations) affect these interactions.
In general, this question is very exciting and worth to investigate. The concept of an altered interactome dependent on specific mutations might explain different phenotypes observed in patients, especially when an RBP with multiple protein and RNA interaction partners is responsible for that.
I have, however, two major concerns with the manuscript that do not favor publication at this state.

1. The central hypothesis of this work is "that R1147W disrupts PUM1's ability to interact with its native partners, which would then lead to de-repression of the targets of these complexes and not just the direct targets of PUM1" lacks experimental evidence supporting the working model. The presented data are rather descriptive and do not support the authors' conclusion.
2. The quality of the presented data fails to provide solid and convincing evidence for their suggested models.

Major points:
1. Regarding the authors aim of unraveling Pum1's role in neurodegenerative diseases, determining brain region specific interactors is important. However, Figure 1 and 2 are redundant. In addition, although the authors tried to reduce the complexity by analyzing different brain areas, these samples still consist of multiple different cell types. It is not clear whether Pum1 and its potential interactors are expressed in all of these cells. Filtering these datasets with existing single cell seq datasets would be important. Moreover, the authors should address post-lysis effects as well to control for the specificity of their interactome study.
2. The importance of Fig.3B does not become clear. The authors already showed these interactions in brain lysate. In respect of the addressed question, HEK cells are not be the appropriate model system. Furthermore, the experiment in Fig.3A (in particular

for the left panel / without RNase) misses important controls. Importantly, a non-binding control is missing to judge specificity. The efficiency of co-IPs can vary a lot between different experiments. If the authors intended to make the point that specific interactions depend on the presence of RNA, the authors need to present quantifications (including standard deviation).
3. The authors put major effort in analyzing steady state levels of the identified Pum1 interactors depending on brain region, sex, and the presence of Pum1 itself. The genetic interaction between different RBPs that is apparently sex- and brain region specific is interesting. However, this point is not well integrated into the story. Instead, the authors jumped to the microRNA machinery. To address this aspect properly, the authors have to perform miRNA seq.
4. The entire Fig.5 is based on published data and subsequent in silico analysis. As these datasets are used from different experimental systems, the conclusions seem to be far-fetched, are therefore not convincing in this context and would need specific experimental validation.
5. Fig.6 has several major problems: (i) in A and B, the blot for GAPDH is only shown for input but not for IPs. (ii) Again, the efficiency of co-IPs can vary a lot between different experiments. If the authors intended to make the point that specific interactions indeed depend on the presence of RNA, the authors need to present quantifications (including standard deviation). (iii) yes, there are changes in Subject1/PADDAS/R114W. But the authors should at least comment on why mutations in Pum1 might reduce interactions. The co-isolation of FMRP AND CNOT1 is NOT affected in my opinion. This could also mean that the interactions between Pum1 and these interactors are enhanced, rather that the others are impaired. (iv) There are major inconsistencies between the inputs in A and B and the corresponding steady state levels in C and D, e.g. CNOT1 in present in equal amounts in A (ctrl. vs. S1) but reduced in C and D; in contrast CNOT1 is reduced in B (ctrl. vs. S3) but present in equal amounts in C and D. In addition, the functional relevance of this altered interaction network remains unclear.
6. The results presented in Fig.7 fit the hypothesis. However, if the authors really wanted to nail down that the altered RBP network due to reduced Pum1 level is causal for the phenotypes observed in patients, they need to establish an rescue assay by overexpression of Pum1.

Referee #2:

Botta and colleagues present a very interesting manuscript evaluating in vivo protein interactions of the Pum1 protein from murine brains. This approach forms a strong foundation and utilizes quantitative technologies to measure Pum1-protein interactions. The authors then test the conservation and importance of these protein-protein binding events in mutations from human patients with Ataxia. This approach, enables the authors to identify changes in Pum1-interacting proteins in Pum1 mutants and includes a detailed analysis of miRNA and protein changes. Using a nice balance of genome wide and molecular biology protocols the authors provide a comprehensive analysis. Although this manuscript covers a lot of experiments, I find the descriptions and discussions accurate and overall it was a pleasure to read.
Based on the high quality data presented, I don't have any major revisions. I do understand that antibodies vary in quality, but some of the IP data presented as western blots in this manuscript was very faint and hard to interpret. This is the only minor issue with this work.
I believe that this research is of high quality and will be of great impact to both the PUM and ATAXIA research community.

Referee #3:

Botta et al in their manuscript entitled "Dosage sensitivity in Pumilio-related phenotype reflects distinct disease mechanisms" reported their systemic characterization of the PUM1 protein complex and the proteins interacting with Pum1 in the mouse brain, and individual regions of the brain in both males and females, hence for the first time establishing a Pum1 interactome. Furthermore, they found that Pum1 interactors and miRNA machinery showed brain region- and sex-specific responses to Pum1 loss; and Pum1 and its RNA-binding protein interactors share many neuronal mRNA targets. The authors then explore the effect of two previously characterized human PUM1 mutations (R1147W) and (T1035S) on Pum1 interacting proteins, showing R1147W, not T1035S mutation, resulted in a reduced level of some of PUM1 interactors and upregulation of their common RNA targets. They further demonstrated such an effect on Pum1 interacting proteins and their shared RNA target expression could be rescued by over-expressing wildtype Pum1 in the R1147 mutant cell line. Hence the authors proposed there are two distinct mechanisms for the two types of mutant PUM1 diseases.

Extending from their previous novel findings of PUM1 mutation-caused diseases, the authors set out to characterize the Pum1 protein complex in the brain and different brain regions and sex. The work was carefully carried out, with extensive and solid data from their LC-MS/MS work and validation. The data will likely be of great value to the field, but there are some concerns, particularly in their interpretation of the data and their proposed model.

Major comments:
1. While the authors presented their data on the protein level change in the interacting proteins of Pum1 in the mutant Pum1 brain, the possibility that some of the interacting proteins could be translationally regulated by Pum1 also exists, especially some interacting proteins PUM2, Cnot1, and Fmr1 contain Pumilio response elements on the 3' UTR of their transcripts. The impact of such a possibility on their model needs to be addressed.

2. Both mild and severe forms of PUM1 mutant patients exhibit different severity in the disease, patients exhibit developmental delay and other defects in addition to the neurological disease like ataxia, could some of the symptoms be caused by mutant PUM1 outside the brain?
3. What is the nature of the Pum1 mouse mutation used in this analysis, and discuss/compare the potential effect of this particular Pum1 mutation with those of human PUM1 mutations?
4. While the loss of PUM1 exhibited a dosage-sensitive effect, it is best to use a complete loss-of-function allele or a mutant allele with the same mutation. It will be much harder to compare a point mutation with a loss of function despite their common effect on the dosage of Pum1 protein.

Minor comments:

Line 27, Pum1 haploinsufficiency disrupts several interactors and regulation of their targets, no evidence presented on such disruption, only reduced level of interactors in Pum1 mutants
Line 26 mild PUM1 loss?
Line 96, please state clearly what constitutes the criteria for putative interactors in this experiment, if the same as the whole brain.
Line123 we were able pull down?
Line 163 but in female null cerebella Fmrp was almost 7% lower (Figure 4A), on the figure 4A, Fmrp in female Pum1-/- cerebellum appears to be more than those in wt?

We thank the reviewers for their comments. Below, we quote the original comments in italics and respond to each in regular font. Where we felt it beneficial to split one comment into several parts, we indicate this with […], but be assured that all reviewer comments are ultimately quoted in full.

Referee 1

In the present manuscript "Dosage sensitivity in Pumilio1-related phenotypes reflects distinct disease mechanisms" by Botta and colleagues aimed at unraveling why differences in expression of the RNA binding protein (RBP) Pumilio1 (Pum1) caused by pathogenic mutations results in clearly different phenotypes. To this end, the authors analyzed the interactome of Pum1 in different brain regions. In addition, the authors investigated how reduced levels of Pum1 (due to pathogenic mutations) affect these interactions.

In general, this question is very exciting and worth to investigate. The concept of an altered interactome dependent on specific mutations might explain different phenotypes observed in patients, especially when an RBP with multiple protein and RNA interaction partners is responsible for that.

I have, however, two major concerns with the manuscript that do not favor publication at this state.

1. The central hypothesis of this work is "that R1147W disrupts PUM1's ability to interact with its native partners, which would then lead to de-repression of the targets of these complexes and not just the direct targets of PUM1" lacks experimental evidence supporting the working model. The presented data are rather descriptive and do not support the authors' conclusion.

2. The quality of the presented data fails to provide solid and convincing evidence for their suggested models.

We are very glad the reviewer finds the question exciting and worthwhile. Their comments prompted us to clarify certain points in the manuscript, as we describe below. We realize there is a tremendous amount of data to think through, and we trust that the revised manuscript makes the story easier to follow.

Major points:
1. Regarding the authors aim of unraveling Pum1's role in neurodegenerative diseases, determining brain region specific interactors is important. However, Figure 1 and 2 are redundant […].

The figures are not actually redundant— **Figure 1** shows the whole-brain interactome whereas **Figure 2** shows the brain-region interactomes— but we considered whether one could be moved to supplementary. We decided it was nicer for readers to see how the interactomes compare.

In addition, although the authors tried to reduce the complexity by analyzing different brain areas, these samples still consist of multiple different cell types. It is not clear whether Pum1 and its potential interactors are expressed in all of these cells. Filtering these datasets with existing single cell seq datasets would be important. […]

We agree with the reviewer that understanding which cell types in the brain express Pum1 will be important and interesting. That is a different project, however: here we are interested in protein interactors, and single cell seq measures RNA, which does not always correlate with protein expression. In fact, this is the first study that seeks to identify interactors, rather than targets, of an RNA-binding protein.

Moreover, as the reviewer him/herself brings up below, published datasets often come from different experimental systems. One of the strengths of this study is that we were able to perform proteomics from

the same brain regions from the same mice of the same age and sex (see **Figure S3,** now **Fig. EV1**).

Moreover, the authors should address post-lysis effects as well to control for the specificity of their interactome study.

We have several lines of evidence that argue against post-lysis effects, and this comment prompted us to clarify this in the manuscript and to summarize the evidence in the opening of the discussion.

First of all, several proteins are expressed at the same levels in different brain regions but interact with PUM1 only in a particular region or two. For example, Pum2 interacts with Pum1 specifically in cortex but not in the other brain regions, despite being more highly expressed in these other regions. The same is true for several other proteins, as we noted in the main text. The fact that repeated experiments turned up the same interactions or lack of interactions in specific brain regions argues strongly against the interaction being an artifact (**Figure S3,** now **Fig. EV1**).

We also went through a great deal of effort to purify recombinant proteins for in vitro studies, even collaborating with an expert biochemist to do so when my own lab failed. We were able to purify Fmrp and Pum2; unfortunately, even our expert collaborators were unable to purify Cnot1 and Ago2 due to the high molecular weight of the first and the tendency of the second to aggregate. (We tried for over a year.) However, we were able to show that Fmrp and Pum2 directly interact with Pum1, ruling out any post-lysis effect with these two interactors (**Figure S4A and B,** now **Fig. EV2A, B**). Furthermore, we then went on to study the <u>functional</u> effects of loss of PUM1 on the behavior of AGO2, FMRP, and the other key RBP interactors, showing consistent sex differences, effects on miRNA and their targets, and so on. The evidence in total is extremely strong.

2. The importance of Fig.3B does not become clear. The authors already showed these interactions in brain lysate. In respect of the addressed question, HEK cells are not be the appropriate model system.[…]

Sorry this wasn't clear. We had to first perform IP experiments in HEK293T cells, with and without RNase treatment, to determine whether the PUM1 interactors behaved the same way in cell type we would later be using for *in vitro* experiments.

Furthermore, the experiment in Fig.3A (in particular for the left panel / without RNase) misses important controls. Importantly, a non-binding control is missing to judge specificity. The efficiency of co-IPs can vary a lot between different experiments. […]

The non-binding controls (to show antibody specificity and efficiency) for **Figure 3A** are presented in **Figure S5A-B** (now **Fig. EV3A,B**) and **Figure S1C** (now **Appendix Fig. S1C**), as detailed below:

1) Regarding <u>antibody specificity</u>: We used Pum1 antibody in samples from Pum1-KO mice (which obviously have no PUM1) and found no interaction (**Figure S5A,** now **Fig. EV3A**), confirming the specificity of the antibody. We also IPed Pum1 and looked for Ago1 and Ago3, which are similar to Ago2 but do not appear in our IP Mass Spec (**Figure S5B,** now **Fig. EV3B**). Furthermore, we co-IPed each Pum1 interactor from WT and Pum1-KO mice with and without RNase treatment and blotted for Pum1 (**Figure S5C,** now **Fig. EV3C**).

2) To test the <u>efficiency of IP</u>, we quantified Pum1 Post-IP before beginning any IP experiment and ensured that our IP can pull down 100% of Pum1 (**Figure S1C**, now **Appendix Fig. S1C**).

If the authors intended to make the point that specific interactions depend on the presence of RNA, the authors need to present quantifications (including standard deviation).

IP experiments are qualitative rather than quantitative. To be RNA-dependent, there must be no interaction after treatment with RNase. See Zhang et al., *Gen. Dev.* 2017 (**Figure 7**), Sternburg et al.,

Scientific Reports 2018 (**Suppl. Fig. 10**), and De Maio et al., *Cell Reports* 2018 (**Figure 1**).

3. The authors put major effort in analyzing steady state levels of the identified Pum1 interactors depending on brain region, sex, and the presence of Pum1 itself. The genetic interaction between different RBPs that is apparently sex- and brain region specific is interesting. However, this point is not well integrated into the story. […]

We found the sex differences by chance observation but were able to use them to demonstrate that there is a functional effect of loss of PUM1 interactions in the brain. This enabled us to move into Ago2/microRNA studies (see below).

Instead, the authors jumped to the microRNA machinery. To address this aspect properly, the authors have to perform miRNA seq.

We actually did! Please see **Figures 4D, E, S8** (now **Appendix Fig. S4**) and **S9** (**Appendix Fig. S5**), which show differentially expressed miRNAs in the cerebellum, where Ago2 has a sex-specific effect. Importantly, we also assessed 49 target genes that were co-bound by the dysregulated miRNAs and confirmed the observed sex-specificity once more.

4. The entire Fig.5 is based on published data and subsequent in silico analysis. As these datasets are used from different experimental systems, the conclusions seem to be far-fetched, are therefore not convincing in this context and would need specific experimental validation.

The reviewer is referring to our bioinformatically overlapping the targets of PUM1 with the targets of the RBP interactors from published datasets, all from mice. It is true that the ages of the mice differed somewhat (a couple of weeks), but this is what was available to analyze—in fact, this makes the results of the overlapping even more impressive. But this in silico analysis was only the beginning of our work: in **Figure 7A**, we experimentally validated shared targets. As we stated in the main text: "Of the 175 targets shared between PUM1, PUM2, AGO2, FMRP, and RBFOX3 (**Table S5,** now **Table EV5**), 54 were expressed in both R1147W fibroblasts and T1035S lymphoblastoid cells. Fifty-one of those were upregulated in the former but not the latter (**Figure 7A**)."

5. Fig.6 has several major problems: (i) in A and B, the blot for GAPDH is only shown for input but not for IPs.[…]

Please note that we are pulling down endogenous proteins, not tagged, so GAPDH cannot be used in IPs. It is not possible to quantify the endogenous levels of proteins that do not interact with your IPed protein, in this case PUM1. We therefore decided to remove GAPDH blots from the inputs to avoid any confusion.

(ii) Again, the efficiency of co-IPs can vary a lot between different experiments. If the authors intended to make the point that specific interactions indeed depend on the presence of RNA, the authors need to present quantifications (including standard deviation).[…]

Figure 6 contains the studies in patient cell lines, where we did not use any RNase treatment. In terms of IP efficiency, we IPed PUM1 and checked for Post-IP in both fibroblasts and lymphoblastoid cell lines and found it to be 100% efficient. **Figures S12A** and **B** (now **Appendix Fig. S8A, B**) show these results. We also add the same blots at longer exposure to better visualize the Pre- and Post-IPs (**Appendix Fig. S8A,** and **B** ***lower panels***).

(iii) yes, there are changes in Subject1/PADDAS/R114W. But the authors should at least comment on why

mutations in Pum1 might reduce interactions.[…]

We hesitate to speculate on the effects of specific mutated residues without structural studies. What we did comment on in the paper is that R1147W exerts a dominant-negative effect over wild-type PUM1.

The co-isolation of FMRP AND CNOT1 is NOT affected in my opinion. This could also mean that the interactions between Pum1 and these interactors are enhanced, rather than the others are impaired.

We did not claim any change in FMRP. To quote our main text: " The mild T1035S variant had no effect on any of these proteins (Figure 6B), but R1147W impaired PUM1 association with AGO2, MOV10, and CNOT1 (Figure 6A)." Consider **Figure 6A** and **D**, which present quantification from Western blot after the IP. CNOT1 is reduced by 50% in both the IP (panel **A**) and in quantification (panel **D**). MOV10, by contrast, disappears in the blot that measures its interaction with PUM1 (panel **A**) but is not reduced in quantity (panel **D**), meaning that it no longer interacts with PUM1 R1147W, but its own levels are unaffected. This is not the case with several other RBPs (see panel **D**).

The reviewer is correct that some interactions with mutant Pum1 could be enhanced, but this is not what we see with the particular interactors we're testing in this manuscript.

(iv) There are major inconsistencies between the inputs in A and B and the corresponding steady state levels in C and D, e.g. CNOT1 in present in equal amounts in A (ctrl. vs. S1) but reduced in C and D; in contrast CNOT1 is reduced in B (ctrl. vs. S3) but present in equal amounts in C and D. In addition, the functional relevance of this altered interaction network remains unclear.

As noted in our response to the preceding comment, Panels **A** and **B** (IPs) are looking at interactions, whereas **C** and **D** (western blots) are quantifying protein levels.

There are several possible explanations for the inconsistencies in the input lanes in **A** and **B**: 1) These are primary fibroblasts and lymphoblastoid cells from human patients, so there is intrinsic variation; 2) The IP protocol and buffer we used are very different from a Western Blot buffer, which is harsher, whereas the IP buffer is intended to preserve the interactions; 3) IP are not meant to be quantitative, whereas western blots can use Gapdh and buffer to extract the entire amount of protein; 4) For a quantitative approach, see **Figure S13A-F** (now **Fig. EV5A-F**) above, where we can precisely quantify the amount of protein regardless of endogenous levels by using tagged proteins.

The functional significance of the altered interaction is demonstrated in **Figure 7**, <u>where target genes shared by Pum1 interactors are upregulated only in the severe disease PADDAS (R114W) and not in the mild PRCA (T1035S)</u>. This evidence is central support for our hypothesis.

6. The results presented in Fig.7 fit the hypothesis. However, if the authors really wanted to nail down that the altered RBP network due to reduced Pum1 level is causal for the phenotypes observed in patients, they need to establish an rescue assay by overexpression of Pum1.

The rescue experiment is in **Figure 7**. We transfected PUM1 WT back into R1147W, rescuing not only the PUM1 interactors but also their shared targets.

To recap, there are four main lines of evidence supporting our hypothesis:

1. Changes in the levels of interactors were found in mouse brain regions, depending on region and sex, when Pum1 was reduced by 50% (Pum1 hets) or was completely absent (Pum1 KO).
2. Expression levels of the partner proteins fell 40-70% in patient cell lines, *despite unchanged mRNA levels.*
3. Studies using MYC- and GST-tagged proteins showed that Ago2 and Cnot1 lose their interaction with Pum1 1147W *in vitro* as well as in patient-derived cells.

4. When we expressed wild-type PUM1 in the patient cell lines with the R1147W mutation, which we believe exerts dominant-negative effect, the levels of the interactors and the shared targets were restored. This confirms that wild-type PUM1 dosage is important in stabilizing the interactors and enabling them to perform their downstream functions.

Together these data persuade us that a 50+% loss of PUM1 disrupts its interactions with its partners, leading PADDAS to be a much more severe phenotype than PRCA (~25% loss of WT Pum1).

Referee 2

Botta and colleagues present a very interesting manuscript evaluating in vivo protein interactions of the Pum1 protein from murine brains. This approach forms a strong foundation and utilizes quantitative technologies to measure Pum1-protein interactions. The authors then test the conservation and importance of these protein-protein binding events in mutations from human patients with Ataxia. This approach, enables the authors to identify changes in Pum1-interacting proteins in Pum1 mutants and includes a detailed analysis of miRNA and protein changes. Using a nice balance of genome wide and molecular biology protocols the authors provide a comprehensive analysis. Although this manuscript covers a lot of experiments, I find the descriptions and discussions accurate and overall it was a pleasure to read.

We deeply appreciate that the reviewer found the manuscript a pleasure to read: we worked extremely hard to make it so!

Based on the high quality data presented, I don't have any major revisions. I do understand that antibodies vary in quality, but some of the IP data presented as western blots in this manuscript was very faint and hard to interpret. This is the only minor issue with this work.

We agree. Sometimes obtaining IP from the mouse brain is difficult, and we must validate them multiple times before selecting the input and IPs from the same blot.

I believe that this research is of high quality and will be of great impact to both the PUM and ATAXIA research community.

Thank you. We sincerely hope so.

Referee 3

Botta et al in their manuscript entitled "Dosage sensitivity in Pumilio-related phenotype reflects distinct disease mechanisms" reported their systemic characterization of the PUM1 protein complex and the proteins interacting with Pum1 in the mouse brain, and individual regions of the brain in both males and females, hence for the first time establishing a Pum1 interactome. Furthermore, they found that Pum1 interactors and miRNA machinery showed brain region- and sex-specific responses to Pum1 loss; and Pum1 and its RNA-binding protein interactors share many neuronal mRNA targets. The authors then explore the effect of two previously characterized human PUM1 mutations (R1147W) and (T1035S) on Pum1 interacting proteins, showing R1147W, not T1035S mutation, resulted in a reduced level of some of PUM1 interactors and upregulation of their common RNA targets. They further demonstrated such an effect on Pum1 interacting proteins and their shared RNA target expression could be rescued by over-expressing wildtype Pum1 in the R1147 mutant cell line. Hence the authors proposed there are two distinct mechanisms for the two types of mutant PUM1 diseases.

Extending from their previous novel findings of PUM1 mutation-caused diseases, the authors set out to characterize the Pum1 protein complex in the brain and different brain regions and sex. The work was carefully carried out, with extensive and solid data from their LC-MS/MS work and validation. The data will likely be of great value to the field, but there are some concerns, particularly in their interpretation of the data and their proposed model.

We thank the reviewer for their positive comments.

Major comments:

1. While the authors presented their data on the protein level change in the interacting proteins of Pum1 in the mutant Pum1 brain, the possibility that some of the interacting proteins could be translationally regulated by Pum1 also exists, especially some interacting proteins PUM2, Cnot1, and Fmr1 contain Pumilio response elements on the 3' UTR of their transcripts. The impact of such a possibility on their model needs to be addressed.

The reviewer is absolutely correct, which is why we tested the RNA levels for each of the PUM1 validated interactors. We found that only Pum2 increased in RNA levels, but not Cnot1, Fmr1, Ago2, Rbfox, or Mov10 (see **Figure S6B** and **S7,** now **Fig. EV4B** and **Appendix Fig. S3**). This holds true for both the entire brain and different brain regions.

2. Both mild and severe forms of PUM1 mutant patients exhibit different severity in the disease, patients exhibit developmental delay and other defects in addition to the neurological disease like ataxia, could some of the symptoms be caused by mutant PUM1 outside the brain?

This is an interesting question. Pum1 is clearly expressed outside the brain, and we might expect infertility in patients with more severe mutations. Most patients we have been able to recruit so far are relatively young, however, and the clinical picture is dominated by symptoms clearly of neurological origin (whether it be ataxia in the mild patients or seizures and various developmental delays in the more severe patients). It will take time for us to learn if the patients (or the mutant mice) have problems in other areas such as immune function, endocrine function, etc.

3. What is the nature of the Pum1 mouse mutation used in this analysis, and discuss/compare the potential effect of this particular Pum1 mutation with those of human PUM1 mutations?

We used Pum1-heterozygous and KO mice in this study to validate PUM1 interactors, and we used patient cells with R1147W or T1035S mutations to assess the effect of those interactors' variants. As shown in **Figure S13E (**now **Fig. EV5**), PUM1 dimerizes and the R1147W destabilizes the WT protein, indicating that R1147W should have more severe effects than Pum1 heterozygosity. We have just gotten R1147W mice and are beginning to assess them, and they do appear more sick than the hets (most notably, they're born much less frequently).

4. While the loss of PUM1 exhibited a dosage-sensitive effect, it is best to use a complete loss-of-function allele or a mutant allele with the same mutation. It will be much harder to compare a point mutation with a loss of function despite their common effect on the dosage of Pum1 protein.

Perhaps the reviewer here is saying that we should differentiate between the two conditions more clearly in the paper, which we are happy to do.

The reviewer is correct that although R1147W causes roughly 50% lower steady-state levels of PUM1 compared to WT, it's not exactly haploinsufficient. It is much less stable (much shorter half life),

and it exerts a dominant-negative effect on WT PUM1. So the phenotype of R1147W mice, which we are only now having in the lab, is actually worse than the phenotype of haploinsufficient mice.

Minor comments:

Line 27, Pum1 haploinsufficiency disrupts several interactors and regulation of their targets, no evidence presented on such disruption, only reduced level of interactors in Pum1 mutants

We can change this in the abstract; we were thinking of the experiments in **Figure 6** with R1147W, but we confused the reader by then saying "we validated these phenomena in patient cell lines" in the next sentence.

Line 26 mild PUM1 loss?

We mean that the mild mutation drops PUM1 levels only about 25%, as opposed to the 50% loss caused by heterozygous deletion, or the slightly greater loss (~55%) seen with R1147W.

Line 96, please state clearly what constitutes the criteria for putative interactors in this experiment, if the same as the whole brain.

We stated this in lines 104-107: "To increase the likelihood that our candidates would prove to be genuine interactors, the proteins had to have at least two unique peptides present in at least five out of six IP-Pum1 samples and also show at least an average >1.5-fold difference between IP-Pum1 and IP-IgG in label-free quantification intensity (LFQ-intensity, see Materials and Methods)." The criteria were the same for interactors in whole brain and brain regions. (Line 138-129: "As before, we selected proteins with at least two unique peptides and an average of >1.5 fold-change between IP-Pum1 and the IP-IgG.")

Line123 we were able pull down?

We cannot find this s in line 123, but "pull down" refers to IP experiments. We will rephrase.

Line 163 but in female null cerebella Fmrp was almost 7% lower (Figure 4A), on the figure 4A, Fmrp in female Pum1-/- cerebellum appears to be more than those in wt?

The reviewer meant 70%, not 7%, but the point stands: thanks to this comment we realized that the blot was accidentally inverted! The quantification was correct, however. We sincerely apologize for our inversion error.

Dear Alessandro,

Thank you for submitting your manuscript for consideration by the EMBO Journal. It has now been seen by three referees whose comments are shown below.

Given the referees' recommendations, I would like to invite you to submit a revised version of the manuscript, addressing the comments of all three reviewers. As discussed, upon cross-commenting, referee three has stated: "If the authors could considerably rephrase their aim, discussion, and conclusions with a clear statement that their current hypothesis on disease mechanism for PADDS should be tested with the knock-in mouse model, I will support the publication of the manuscript without the inclusion of the mouse data." Therefore, if you make the appropriate changes to the text, the inclusion of this data is not required in this manuscript.

Thank you for the opportunity to consider your work for publication. I look forward to your revision.

Yours sincerely,

Kelly

Kelly M Anderson, PhD
Editor
The EMBO Journal
k.anderson@embojournal.org

Instructions for preparing your revised manuscript:

Please make sure you upload a letter of response to the referees' comments together with the revised manuscript.

Please also check that the title and abstract of the manuscript are brief, yet explicit, even to non-specialists.

When assembling figures, please refer to our figure preparation guideline in order to ensure proper formatting and readability in print as well as on screen:
https://bit.ly/EMBOPressFigurePreparationGuideline
See also guidelines for figure legends: https://www.embopress.org/page/journal/14602075/authorguide#figureformat

At EMBO Press we ask authors to provide source data for the main manuscript figures. Our source data coordinator will contact you to discuss which figure panels we would need source data for and will also provide you with helpful tips on how to upload and organize the files.

IMPORTANT: When you send the revision we will require
- a point-by-point response to the referees' comments, with a detailed description of the changes made (as a word file).
- a word file of the manuscript text.
- individual production quality figure files (one file per figure)
- a complete author checklist, which you can download from our author guidelines (https://www.embopress.org/page/journal/14602075/authorguide).
- Expanded View files (replacing Supplementary Information)

Please see out instructions to authors
https://www.embopress.org/page/journal/14602075/authorguide#expandedview

Please remember: Digital image enhancement is acceptable practice, as long as it accurately represents the original data and conforms to community standards. If a figure has been subjected to significant electronic manipulation, this must be noted in the figure legend or in the 'Materials and Methods' section. The editors reserve the right to request original versions of figures and the original images that were used to assemble the figure.

Further information is available in our Guide For Authors: https://www.embopress.org/page/journal/14602075/authorguide

We realize that it is difficult to revise to a specific deadline. In the interest of protecting the conceptual advance provided by the work, we recommend a revision within 3 months (21st May 2023). Please discuss the revision progress ahead of this time with the editor if you require more time to complete the revisions.

Referee #1:

In the revised version of this Pum1 manuscript by Dr. Gennarino et al., the authors added additional information. As outlined in the initial review, the scientific question is very exciting and an important topic to investigate. The presented concept of an altered interactome dependent on specific mutations might indeed explain different phenotypes observed in patients, especially when an RBP with multiple protein and RNA interaction partners is responsible for that. However, the quality of (at least some of) the (biochemical) data is in my opinion not as complete and convincing as it should be and some of the conclusions might not be adequate at this time. Overall, I find the study to be interesting, the results promising and certainly important for a broad community. In general, I am not against publication, but want to express at this stage my scientific concerns. In my opinion, the central hypothesis of this work is "that R1147W disrupts PUM1's ability to interact with its native partners, which would then lead to de-repression of the targets of these complexes and not just the direct targets of PUM1" lacks conclusive experimental evidence clearly and strongly supporting the current working model.
Absolutely essential for the title and the story is Fig. 6 (see my initial review). Here, a few key points were suggested to the authors, but were not addressed or really taken into account. First, the Western blots for GADPH were lacking in the initial submission (initial point 5). Of course, GADPH is very likely not to be detected in the IPs, but the 'empty membrane' has to be included (omitting the data as it is now is not an option). Second, it is simply not correct to state that IPs cannot be quantified. There is ample evidence from many leading biochemical labs that one can indeed make a serious effort to quantify 'the co-IP efficiency compared to input and control' and get a first objective impression of the presented results. In my opinion, Fig. 6 needs additional changes in order to meet the quality level of EMBO J. First, the CNOT1 reduction seen in the IP has to be placed in the context of the drastically reduced Pum1 signal (approx. 10%, see panel A in Fig. 6). Most likely, it is rather somewhat increased compared to control conditions. Third, there are inconsistencies within the various blots of Fig. 6, e.g. (i) panel A, CNOT1 input signal control vs patient S1 is virtually identical. However, the authors detect a reduction in CNOT1 levels, controls vs S1, panels DE; (ii) FMRP is reduced in the input signals control vs patient S3 (panel B) but identical in panel C. It might be that the authors are right in the response reg. the intrinsic variations using different cellular systems (see rebuttal), however, this possibility needs to be experimentally validated. Detergents, indeed, can have an effect on lysis and protein complex stability, but this of course applies to both wt and patient samples within one experiment.

Referee #2:

The authors have addressed my comments and I find this research highly interesting; I think it will significantly contribute to the field.

Referee #3:

In their revised manuscript entitled "Dosage sensitivity to Pumilio 1 variants reflects distinct molecular mechanisms", Botta et al. have responded to comments from the reviewers to some extent, but their response to the concerns about their central hypothesis and the supporting data is less satisfactory.

There are at least three scenarios that PADDAS mutation could cause the reduction of interactor proteins of PUM1, (AGO2, CNOT1, MOV10) while PRCS mutation does not.
PADDAS mutant protein is not stable, leading to a significant reduction of PUM1 protein and subsequently loss of its interacting proteins
PADDAS mutation is a dominant-negative mutation, that severely impacts its interaction with its interacting proteins
PADDAS mutation affects the translation of PUM1 interacting proteins without affecting their mRNA expression, leading to a significantly reduced amount of interacting proteins and hence less amount of interacting proteins in PUM1 pulled-down proteins.
The authors used patient-derived fibroblast cells for PADDAS and lymphoblastoid cells for PRCS mutation and found that several interacting proteins decreased in PADDAS fibroblast cells but not in PRCS lymphoblastoid cell lines. This is really not the best system to determine the difference between two genetic mutations conclusively due to difference in cell lines and the presence of wildtype PUM1. While the authors argue that PADDAS may be a dominant-negative allele, the fact that overexpression of wt-PUM1 restores the expression levels of PUM1 interactors argues otherwise.

If the authors have already acquired PADDAS knock-in mice as they claim in their rebuttal, these mice might be best to address the nature of this PADDAS, especially in comparison with the loss-of-function allele of mouse Pum1 mutation. It should not be difficult to directly test their hypothesis by comparing the expression of PUM1-interacting proteins (AGO2, FMRP,CNOT1 and MOV10) and the expression of their common targets with those of Pum1 knockout heterozygotes.

Current data does not provide compelling support for their central hypothesis, although the authors have generated extensive data on PUM1 interacting proteome and interesting regional and sex differences in those interacting proteins in absence of Pum1, supporting potentially important roles of those interacting proteins.

The authors also did not address several of my specific concerns satisfactorily either.

1. While the authors presented their data on the protein level change in the interacting proteins of Pum1 in the mutant Pum1 brain, the possibility that some of the interacting proteins could be translationally regulated by Pum1 also exists, especially some interacting proteins PUM2, Cnot1, and Fmr1 contain Pumilio response elements on the 3' UTR of their transcripts. The impact of such a possibility on their the model needs to be addressed.
The authors only consider the RNA expression change for those interactor proteins but did not consider the scenario with even unchanged RNA expression for Cnot1, Fmr1, Ago2 or Rbfox, their protein level could change due to the effect of Pumilio1 mutation on their translation. Such a possibility could contribute to the change in protein expression of PUM1 interactors they observed and need to be considered.

2. What is the nature of the Pum1 mouse mutation used in this analysis, and discuss/compare the
The potential effect of this particular Pum1 mutation with those of human PUM1 mutations?
The question here is to ask what type of Pum1 mutation in the Pum1 knockout mice is, is it a point mutation or deletion in RNA binding domain or other parts of Pum1?
While it is not trivial to produce interacting proteome of Pum1 in mouse brain and in the different regions of brains, and interestingly they also found some sex-specific differences in the interacting proteome of PUM1 in various brain regions, but there was a leap in transition to the following parts of the manuscript, or not well integrated as the other referee suggested, where the authors went ahead to compare the effect of the two different human PUM1 mutations on those interacting proteins. The second part of the manuscript presented the data in support of their central hypothesis for two distinct mechanisms in the two subtypes of PUM1-caused diseases using patient-derived cell lines and overexpression in the cell line.

3. The authors established PUM1 interactors using mouse models but tested if protein expression of those interactors was disrupted by two PUM1 mutations in the patient-derived cell lines, without showing such interactions are conserved in human brain tissues.

4. The title of the manuscript is awkward and confusing, maybe , Pumilio1 interactome and their contribution to the distinct pathogenic mechanisms of human PUMILIO1 subtype mutations

Minor concerns:
-Line123 we were able pull down? Should it be changed to "we were able to pull down?"
-The first letter of Western blot should be capitalized
-Line 134, strongly tended to aggregate? strong tendency to aggregate
-Line 165 Pum1 interacts with Pum2, Fmrp, Ago2 and Cnot1 prior to binding RNA, RNase treatment experiment only supports such interaction is independent of RNA, not necessarily prior to
-Line 82 two different mechanisms ? two different pathogenic mechanisms
-Line 169 If Pum1 is an important interactor for these six RBPs, loss of Pum1 should affect their abundance or function? is it true that loss of an interacting protein will always affect the abundance of the partner protein? or even function?

We thank all three Reviewers for finding the work both important and interesting, and also for their patience in clarifying their concerns in order to help us better revise the paper. We realized we were insufficiently clear that this paper is not about elucidating *disease pathogenesis*, which will require comparison of PRCA and PADDAS mouse models with intensive characterization of the mice at the behavioral and pathogenic levels and examination of the various brain regions for a much larger set of interactors and targets. Rather, it is fundamentally about *how to understand the function of an RBP*, and secondarily, about challenging the assumption that dosage effects are always linear. We open the paper with how to understand RBP function, and we closed the Discussion with how to understand RBP function. This work doesn't trace the path to PADDAS/PRCA pathogenesis, nor was it meant to.

This paper is the first study to map the interactors of an RBP from the mouse brain and to show that interactors matter, not just targets. This is the first step, albeit a large and crucial one, to lay the groundwork for pathogenesis studies in mice. We now spell out that definitive proof about the different pathogenic pathways for PADDAS and PRCA will require animal modeling and many further studies.

Referee 1:

In the revised version of this Pum1 manuscript by Dr. Gennarino et al., the authors added additional information. As outlined in the initial review, the scientific question is very exciting and an important topic to investigate. The presented concept of an altered interactome dependent on specific mutations might indeed explain different phenotypes observed in patients, especially when an RBP with multiple protein and RNA interaction partners is responsible for that. However, the quality of (at least some of) the (biochemical) data is in my opinion not as complete and convincing as it should be and some of the conclusions might not be adequate at this time. Overall, I find the study to be interesting, the results promising and certainly important for a broad community. In general, I am not against publication, but want to express at this stage my scientific concerns.

We appreciate the reviewer's appreciation of the work's strengths as well as their expressed concerns.

In my opinion, the central hypothesis of this work is "that R1147W disrupts PUM1's ability to interact with its native partners, which would then lead to de-repression of the targets of these complexes and not just the direct targets of PUM1" lacks conclusive experimental evidence clearly and strongly supporting the current working model. Absolutely essential for the title and the story is Fig. 6 (see my initial review). Here, a few key points were suggested to the authors, but were not addressed or really taken into account. First, the Western blots for GADPH were lacking in the initial submission (initial point 5). Of course, GADPH is very likely not to be detected in the IPs, but the 'empty membrane' has to be included (omitting the data as it is now is not an option). Second, it is simply not correct to state that IPs cannot be quantified. There is ample evidence from many leading biochemical labs that one can indeed make a serious effort to quantify 'the co-IP efficiency compared to input and control' and get a first objective impression of the presented results.

We should have explained ourselves better in the first response. It is true that GAPDH will be identified in the protein lysates (input) but not in the IP. We had decided to remove GAPDH from the main Fig 6 before our first submission because patient cell lines have so much biological variability, and in our first rebuttal we cited different manuscripts showing that established labs also do not use GAPDH for IPs in biological samples. Notice that in Fig 6A-B, the inputs from Ctrl and S1, S2 and S3 vary quite a bit: sometimes the inputs from S1, S2 and S3 look very different from Ctrl (see PUM1, FMRP and MOV10), while AGO2 and CNOT1 look more similar—but all are normalized to GAPDH for the quantification (which we have put back into Fig 6).

This biological variability is why we performed the experiments in HEK293T cells using tagged proteins (and GAPDH!) that we show in Fig EV5. But we can understand why the reviewer found the submitted version of Fig 6 less than persuasive, and we have provided the quantification s/he requests.

The next several comments of Reviewer 1 center around the same basic issue, but we break up the original comment to be better able to answer specific points. Basically, it all comes down to variability in

biological samples and to important differences between the methods used in IP and western blot experiments.

In my opinion, Fig. 6 needs additional changes in order to meet the quality level of EMBO J. First, the CNOT1 reduction seen in the IP has to be placed in the context of the drastically reduced Pum1 signal (approx. 10%, see panel A in Fig. 6). Most likely, it is rather somewhat increased compared to control conditions.

The reviewer is correct that the CNOT1 reduction must be interpreted in the context of the reduced PUM1 signal—but because we didn't include quantification, the reviewer gauges the reduction to be more severe (10% of WT) than it actually is (around 50% of WT).

In the revised Fig 6, we put back the GAPDH and present the quantification relative to PUM1 below the blots. (We repeated the experiment at least 6 times to get the quantification and error bars.) PUM1-R1147W in S1 is 50% of WT, not 10%—but is normalized to 1. CNOT1 is reduced by about 31% (compared to the normalized PUM1), both in the patient cell lines (Fig 6A) and HEK293T (Fig EV 5). The ratio of CNOT1 in S1 compared to the input is lower than that of control. See the Methods for more detailed explanation of quantification as well as revised Fig 6 and the raw data.

Third, there are inconsistencies within the various blots of Fig. 6, e.g. (i) panel A, CNOT1 input signal control vs patient S1 is virtually identical. However, the authors detect a reduction in CNOT1 levels, controls vs S1, panels DE;

Panels A and B are IPs, Panel C and D are Western blots. See our response below for further comment on why these experiments cannot be compared directly.

(ii) FMRP is reduced in the input signals control vs patient S3 (panel B) but identical in panel C. It might be that the authors are right in the response reg. the intrinsic variations using different cellular systems (see rebuttal), however, this possibility needs to be experimentally validated. Detergents, indeed, can have an effect on lysis and protein complex stability, but this of course applies to both wt and patient samples within one experiment.

Again, IP and WB are two distinct experiments. As the reviewer notes, western blots employ a much harsher detergent to extract as many proteins as possible. The IP lysate cannot be too harsh, lest the antibody fail. For example, RIPA with 0.1% SDS is used to extract proteins for WB, while no SDS is used for the IP buffer. Briefly, while we cannot extract 100% of proteins with the IP buffer, we can pull down the entire amount of extracted proteins since we used an amount of antibody (3ug to 5 ug) which is much higher than the total amount of extracted proteins. This also suggests why IP experiments are not truly quantitative, and there can be variability in the inputs. Please see **Figure EV5** where we validated the same findings form patient-derived cell lines.

Referee 2:
The authors have addressed my comments and I find this research highly interesting; I think it will significantly contribute to the field.

We thank the reviewer for their interest in the work.

Referee 3:
In their revised manuscript entitled "Dosage sensitivity to Pumilio 1 variants reflects distinct molecular mechanisms", Botta et al. have responded to comments from the reviewers to some extent, but their response to the concerns about their central hypothesis and the supporting data is less satisfactory.

As we state in the preface to this response, we acknowledge that we have not proven the two-pronged hypothesis at the level of disease pathogenesis in animal models of PRCA and PADDAS. Rather, we show that T1035S and R1147W have different effects at the molecular level, in vitro, in patient cells, and in *Pum1* heterozygous and knockout mice. We have revised the manuscript to make this even more clear.

There are at least three scenarios that PADDAS mutation could cause the reduction of interactor proteins of PUM1, (AGO2, CNOT1, MOV10) while PRCS mutation does not.

- PADDAS mutant protein is not stable, leading to a significant reduction of PUM1 protein and subsequently loss of its interacting proteins

Correct: this would be consistent with the fact that R1147W (50% loss of PUM1) causes a more severe phenotype (closer to the KO) than one would expect from the PUM1 het mice.

- PADDAS mutation is a dominant-negative mutation, that severely impacts its interaction with its interacting proteins

Correct: based on **Figure 6**, and **Figure EV5** we believe this is the case.

-PADDAS mutation affects the translation of PUM1 interacting proteins without affecting their mRNA expression, leading to a significantly reduced amount of interacting proteins and hence less amount of interacting proteins in PUM1 pulled-down proteins.

This could be a possibility, but molecularly it ends up in the same place: the PADDAS but not the PRCA mutation alters the expression of interacting proteins.

The authors used patient-derived fibroblast cells for PADDAS and lymphoblastoid cells for PRCS mutation and found that several interacting proteins decreased in PADDAS fibroblast cells but not in PRCS lymphoblastoid cell lines. This is really not the best system to determine the difference between two genetic mutations conclusively due to difference in cell lines and the presence of wildtype PUM1.

The reviewer is correct; we used what we had. We have tried very hard for several years to procure either lymphoblasts from R1147 patients or fibroblasts from T1035S patients, but we've had no luck. This is why we repeated the experiment in HEK293T cells with tagged proteins, and the results strongly support the patient-derived cell line experiments. See **Figure EV5.** Thus, a limitation of the study perversely becomes a strength: the fact that the behavior of interactors and their targets are so consistent across different cell types (insofar as we identified interactors and targets that were common to both), lends support to the notion that these results are real.

While the authors argue that PADDAS may be a dominant-negative allele, the fact that overexpression of wt-PUM1 restores the expression levels of PUM1 interactors argues otherwise.

We do not quite follow the reviewer's train of thought here. The over-expression of PUM1 is high enough to overcome the effect of R1147W. See **Appendix Figure 8C.**

If the authors have already acquired PADDAS knock-in mice as they claim in their rebuttal, these mice might be best to address the nature of this PADDAS, especially in comparison with the loss-of-function allele of mouse Pum1 mutation. It should not be difficult to directly test their hypothesis by comparing the expression of PUM1-interacting proteins (AGO2, FMRP, CNOT1 and MOV10) and the expression of their common targets with those of Pum1 knockout heterozygotes. Current data does not provide compelling support for their central hypothesis, although the authors have generated extensive data on PUM1 interacting proteome and interesting regional and sex differences in those interacting proteins in

absence of Pum1, supporting potentially important roles of those interacting proteins.

We appreciate this comment, because it helped us realize that the Reviewer was expecting (hoping?) this paper would explore the pathogenesis of PRCA and PADDAS. We do have R1147W mice, but we will also need T1035S mice, which have been very tricky to generate. As noted above in our 'preface' to our point-by-point response, characterizing two mouse lines, examining the interactors and targets in different brain regions and sexes, etc., will take a lot more work (and another grant to support it!), and there is no way so much data could be packed into one journal article. We have, however, revised the text to make it clear that we are looking at difference between the two mutants and not attempting pathogenesis studies in this particular manuscript.

At present, we can only emphasize that the experiment in **Figure EV5** using tagged constructs confirms that R1147W destabilizes AGO2 and CNOT1, whereas T1035S does not.

The authors also did not address several of my specific concerns satisfactorily either.

1. While the authors presented their data on the protein level change in the interacting proteins of Pum1 in the mutant Pum1 brain, the possibility that some of the interacting proteins could be translationally regulated by Pum1 also exists, especially some interacting proteins PUM2, Cnot1, and Fmr1 contain Pumilio response elements on the 3' UTR of their transcripts. The impact of such a possibility on their the model needs to be addressed. The authors only consider the RNA expression change for those interactor proteins but did not consider the scenario with even unchanged RNA expression for Cnot1, Fmr1, Ago2 or Rbfox, their protein level could change due to the effect of Pumilio1 mutation on their translation. Such a possibility could contribute to the change in protein expression of PUM1 interactors they observed and need to be considered.

If PUM1 translationally regulates some of these proteins, we would expect the PRCA mutation, which cannot bind RNA, to be unable to regulate them (i.e., their levels should go up). But we do not find that the levels of any of these proteins are altered in PRCA lines.

The PADDAS mutation is able to bind Pumilio Response Elements (PREs) in target proteins (see **Appendix Figure S8C**)—so it can bind Fmr1, Pum2, and Cnot1—but only Ago2 and Cnot1 are destabilized, and Ago2 does not contain a PRE. It is possible that R1147W binds to proteins that could, if dysregulated, change the translation of some of those proteins; this is an avenue of investigation that is worth pursuing in the future, but it does not fundamentally alter the picture here, that only the PADDAS mutation changes the expression of (some) interactors.

2. What is the nature of the Pum1 mouse mutation used in this analysis, and discuss/compare the potential effect of this particular Pum1 mutation with those of human PUM1 mutations? The question here is to ask what type of Pum1 mutation in the Pum1 knockout mice is, is it a point mutation or deletion in RNA binding domain or other parts of Pum1?

We will be sure to clarify this in the text. Briefly, the PUM1 knockout mice were generated by crossing heterozygous Pum1 mice (Chen et al 2012):

> "To understand the role of Pum1 in the mouse germline, we knocked out the Pum1 gene. To circumvent possible embryonic lethality of global Pum1 deficiency, we started with a conditional knockout (cKO) strategy. A Pum1 allele with Exons 8 to 9 flanked by LoxP sites was generated by targeting wild-type Pum1 allele in mouse embryonic stem cells (Figure S1B). Mice on a B6/129 background that carry this allele were generated by the University of Connecticut Gene Targeting and Transgenic Facility. $Pum1^{flox/flox}$ mice were mated with a VASA-cre line in which Cre expression is confined to the gonads [1]. Pum1 testis cKO males ($Pum1^{-/flox}$;VASA-cre/+) thus generated were crossed with wild-type females. In this mating, the Pum1-null allele is +/- transmitted through the male germline and thus the resulting offspring were globally Pum1.

Self-cross of $Pum1^{+/-}$ mice gave rise to Pum1 global KO ($Pum1^{-/-}$) mice and also maintained this strain."

(Their figure S1D shows that they actually deleted exons 8-10.) Deleting the exons causes RNA decay. Not only did Chen et al. verifiy a lack of protein in the Pum1 knockout mice, but we re-verified this absence of protein (Gennarino et al., *Cell* 2015) by both ISH and western blot. See Figure S2A and C in that paper.

While it is not trivial to produce interacting proteome of Pum1 in mouse brain and in the different regions of brains, and interestingly they also found some sex-specific differences in the interacting proteome of PUM1 in various brain regions, but there was a leap in transition to the following parts of the manuscript, or not well integrated as the other referee suggested, where the authors went ahead to compare the effect of the two different human PUM1 mutations on those interacting proteins. The second part of the manuscript presented the data in support of their central hypothesis for two distinct mechanisms in the two subtypes of PUM1-caused diseases using patient-derived cell lines and overexpression in the cell line.

We hope our revised text is clearer.

3. The authors established PUM1 interactors using mouse models but tested if protein expression of those interactors was disrupted by two PUM1 mutations in the patient-derived cell lines, without showing such interactions are conserved in human brain tissues.

It is unlikely that we will ever be able to procure human brain tissue to test such interactions... maybe the referee meant mouse brain? Please see responses above about the quantity of work it will take to articulate the pathogenetic pathways in PRCA and PADDAS mouse models.

4. The title of the manuscript is awkward and confusing, maybe, Pumilio1 interactome and their contribution to the distinct pathogenic mechanisms of human PUMILIO1 subtype mutations

The title is confusing only if the reader expects the paper to be about disease pathogenesis, which is precisely what we are trying to avoid!

Minor concerns:
-Line123 we were able pull down? Should it be changed to "we were able to pull down?"

Thank you.

-The first letter of Western blot should be capitalized

Actually, not so. Only Southern blot is capitalized, because it's named after Edwin Southern. See, for example, this paper on the topic: https://www.ncbi.nlm.nih.gov/pmc/articles/PMC5240824/

-Line 134, strongly tended to aggregate? strong tendency to aggregate

Thank you.

-Line 165 Pum1 interacts with Pum2, Fmrp, Ago2 and Cnot1 prior to binding RNA, RNase treatment experiment only supports such interaction is independent of RNA, not necessarily prior to

Good point.

-Line 82 two different mechanisms ? two different pathogenic mechanisms

We’re deliberately staying away from claims about disease pathogenesis.

-Line 169 If Pum1 is an important interactor for these six RBPs, loss of Pum1 should affect their abundance or function? is it true that loss of an interacting protein will always affect the abundance of the partner protein? or even function?

Not invariably so, but often enough that it would be very surprising if there were no such effect.

2nd Revision - Editorial Decision 14th Mar 2023

Dear Alessandro,

Congratulations on an excellent manuscript, I am pleased to inform you that your manuscript has been accepted for publication in The EMBO Journal. It has been a pleasure to work with you to get this to the acceptance stage.

Thank you for providing detailed source data and for your comprehensive response to the referee concerns. Specifically, as requested by the referees, your revised version emphasizes that the focus of this study is not to establish the pathogenesis of PADDAS and that as you now point out in your discussion "elucidating the pathogenesis of these diseases will require animal models". Your revised version makes more clear that the focus of your study is to demonstrate a strategy to understand the function of an RNA-binding protein and to challenge the assumption that dosage effects are linear, which we find to be of great importance. I appreciate that you have added the suggested updates to Figure 6, which now include GapdH in A and B and quantifications as requested. Regarding the differences in IP and western sample data: after carefully reviewing your methods, we find that only the westerns should be comparable across samples as you have quantified protein and loaded the same amount of protein into each lane. In the IP, the amount of protein loaded would be variable depending on a variety of factors in generating the sample and would only be comparable after normalizing to the input value. Therefore, we are satisfied with your argument that IP samples are not comparable across samples.

I will begin the final checks on your manuscript before submitting to the publisher next week. Once at the publisher, it will take about 3 weeks for your manuscript to be published online. As a reminder, the entire review process, including referee concerns and your point-by-point response will be available to readers.

I will be in touch throughout the final editorial process until publication. In the meantime, I hope you find time to celebrate!

Kind regards,
Kelly

Kelly M Anderson, PhD
Editor
The EMBO Journal
k.anderson@embojournal.org

Please note that it is EMBO Journal policy for the transcript of the editorial process (containing referee reports and your response letter) to be published as an online supplement to each paper. If you do NOT want this, you will need to inform the Editorial Office via email immediately. More information is available here:
https://www.embopress.org/page/journal/14602075/authorguide#transparentprocess

Your manuscript will be processed for publication in the journal by EMBO Press. Manuscripts in the PDF and electronic editions of The EMBO Journal will be copy edited, and you will be provided with page proofs prior to publication. Please note that supplementary information is not included in the proofs.

You will be contacted by Wiley Author Services to complete licensing and payment information. The required 'Page Charges Authorization Form' is available here: https://www.embopress.org/pb-assets/embo-site/tej_apc.pdf - please download and complete the form and return to embopressproduction@wiley.com

EMBO Press participates in many Publish and Read agreements that allow authors to publish Open Access with reduced/no publication charges. Check your eligibility: https://authorservices.wiley.com/author-resources/Journal-Authors/open-access/affiliation-policies-payments/index.html

Should you be planning a Press Release on your article, please get in contact with embojournal@wiley.com as early as possible, in order to coordinate publication and release dates.

** Click here to be directed to your login page: https://emboj.msubmit.net

EMBO Press Author Checklist

Corresponding Author Name: Vincenzo A. Gennarino	
Journal Submitted to: EMBO J	
Manuscript Number: EMBOJ-2022-112721	

USEFUL LINKS FOR COMPLETING THIS FORM
The EMBO Journal - Author Guidelines
EMBO Reports - Author Guidelines
Molecular Systems Biology - Author Guidelines
EMBO Molecular Medicine - Author Guidelines

Reporting Checklist for Life Science Articles (updated January
This checklist is adapted from Materials Design Analysis Reporting (MDAR) Checklist for Authors. MDAR establishes a minimum set of requirements in transparent reporting in the life sciences (see Statement of Task: 10.31222/osf.io/9sm4x). Please follow the journal's guidelines in preparing your
Please note that a copy of this checklist will be published alongside your article.

Abridged guidelines for figures
1. Data
The data shown in figures should satisfy the following conditions:
- the data were obtained and processed according to the field's best practice and are presented to reflect the results of the experiments in an accurate and unbiased manner.
- ideally, figure panels should include only measurements that are directly comparable to each other and obtained with the same assay.
- plots include clearly labeled error bars for independent experiments and sample sizes. Unless justified, error bars should not be shown for technical
- if n<5, the individual data points from each experiment should be plotted. Any statistical test employed should be justified.
- Source Data should be included to report the data underlying figures according to the guidelines set out in the authorship guidelines on Data

2. Captions
Each figure caption should contain the following information, for each panel where they are relevant:
- a specification of the experimental system investigated (eg cell line, species name).
- the assay(s) and method(s) used to carry out the reported observations and measurements.
- an explicit mention of the biological and chemical entity(ies) that are being measured.
- an explicit mention of the biological and chemical entity(ies) that are altered/varied/perturbed in a controlled manner.
- the exact sample size (n) for each experimental group/condition, given as a number, not a range;
- a description of the sample collection allowing the reader to understand whether the samples represent technical or biological replicates (including how many animals, litters, cultures, etc.).
- a statement of how many times the experiment shown was independently replicated in the laboratory.
- definitions of statistical methods and measures:
  - common tests, such as t-test (please specify whether paired vs. unpaired), simple χ2 tests, Wilcoxon and Mann-Whitney tests, can be unambiguously identified by name only, but more complex techniques should be described in the methods section;
  - are tests one-sided or two-sided?
  - are there adjustments for multiple comparisons?
  - exact statistical test results, e.g., P values = x but not P values < x;
  - definition of 'center values' as median or average;
  - definition of error bars as s.d. or s.e.m.

Please complete ALL of the questions below.
Select "Not Applicable" only when the requested information is not relevant for your study.

Materials

Newly Created Materials	Information included in the manuscript?	In which section is the information available? (Reagents and Tools Table, Materials and Methods, Figures, Data Availability Section)
New materials and reagents need to be available; do any restrictions apply?	Yes	Materials and Methods

Antibodies	Information included in the manuscript?	In which section is the information available? (Reagents and Tools Table, Materials and Methods, Figures, Data Availability Section)
For **antibodies** provide the following information:<br>- Commercial antibodies: RRID (if possible) or supplier name, catalogue number and or/clone number<br>- Non-commercial: RRID or citation	Yes	Materials and Methods

DNA and RNA sequences	Information included in the manuscript?	In which section is the information available? (Reagents and Tools Table, Materials and Methods, Figures, Data Availability Section)
**Short novel DNA or RNA including primers, probes**: provide the sequences.	Yes	Materials and Methods

Cell materials	Information included in the manuscript?	In which section is the information available? (Reagents and Tools Table, Materials and Methods, Figures, Data Availability Section)
**Cell lines:** Provide species information, strain. Provide accession number in repository **OR** supplier name, catalog number, clone number, and/**OR** RRID.	Yes	Materials and Methods
**Primary cultures:** Provide species, strain, sex of origin, genetic modification status.	Not Applicable	
Report if the cell lines were recently **authenticated** (e.g., by STR profiling) and tested for mycoplasma contamination.	Yes	Materials and Methods

Experimental animals	Information included in the manuscript?	In which section is the information available? (Reagents and Tools Table, Materials and Methods, Figures, Data Availability Section)
**Laboratory animals or Model organisms:** Provide species, strain, sex, age, genetic modification status. Provide accession number in repository **OR** supplier name, catalog number, clone number, **OR** RRID.	Yes	Materials and Methods
**Animal observed in or captured from the field:** Provide species, sex, and age where possible.	Not Applicable	
Please detail **housing and husbandry conditions**.	Yes	Materials and Methods

Plants and microbes	Information included in the manuscript?	In which section is the information available? (Reagents and Tools Table, Materials and Methods, Figures, Data Availability Section)
**Plants:** provide species and strain, ecotype and cultivar where relevant, unique accession number if available, and source (including location for collected wild specimens).	Not Applicable	
**Microbes:** provide species and strain, unique accession number if available, and source.	Not Applicable	

Human research participants	Information included in the manuscript?	In which section is the information available? (Reagents and Tools Table, Materials and Methods, Figures, Data Availability Section)
If collected and within the bounds of privacy constraints report on age, sex and gender or ethnicity for all study participants.	Not Applicable	

Core facilities	Information included in the manuscript?	In which section is the information available? (Reagents and Tools Table, Materials and Methods, Figures, Data Availability Section)
If your work benefited from core facilities, was their service mentioned in the acknowledgments section?	Yes	Materials and Methods

Design

Study protocol	Information included in the manuscript?	In which section is the information available? (Reagents and Tools Table, Materials and Methods, Figures, Data Availability Section)

If study protocol has been **pre-registered, provide DOI in the manuscript**. For clinical trials, provide the trial registration number **OR** cite DOI.	Not Applicable	
Report the **clinical trial registration number** (at ClinicalTrials.gov or equivalent), where applicable.	Not Applicable	

Laboratory protocol	Information included in the manuscript?	In which section is the information available? (Reagents and Tools Table, Materials and Methods, Figures, Data Availability Section)
Provide DOI OR other citation details if **external detailed step-by-step protocols** are available.	Not Applicable	

Experimental study design and statistics	Information included in the manuscript?	In which section is the information available? (Reagents and Tools Table, Materials and Methods, Figures, Data Availability Section)
Include a statement about **sample size** estimate even if no statistical methods were used.	Yes	Materials and Methods (Software and statistical analysis)
Were any steps taken to minimize the effects of subjective bias when allocating animals/samples to treatment (e.g. **randomization procedure**)? If yes, have they been described?	Yes	Materials and Methods (Experimental design)
Include a statement about **blinding** even if no blinding was done.	Yes	Materials and Methods (Experimental design)
Describe **inclusion/exclusion criteria** if samples or animals were excluded from the analysis. Were the criteria pre-established?<br><br>If sample or data points were omitted from analysis, report if this was due to attrition or intentional exclusion and provide justification.	Not Applicable	
For every figure, are **statistical tests** justified as appropriate? Do the data meet the assumptions of the tests (e.g., normal distribution)? Describe any methods used to assess it. Is there an estimate of variation within each group of data? Is the variance similar between the groups that are being statistically compared?	Yes	Materials and Methods (Software and statistical analysis), Figure legends

Sample definition and in-laboratory replication	Information included in the manuscript?	In which section is the information available? (Reagents and Tools Table, Materials and Methods, Figures, Data Availability Section)
In the figure legends: state number of times the experiment was **replicated** in laboratory.	Yes	Figures Legends
In the figure legends: define whether data describe **technical or biological replicates.**	Yes	Figures Legends

Ethics

Ethics	Information included in the manuscript?	In which section is the information available? (Reagents and Tools Table, Materials and Methods, Figures, Data Availability Section)
Studies involving **human participants**: State details of **authority granting ethics approval** (IRB or equivalent committee(s), provide reference number for approval.	Yes	Materials and Methods (Study approval)
Studies involving **human participants**: Include a statement confirming that **informed consent** was obtained from all subjects and that the experiments conformed to the principles set out in the WMA Declaration of Helsinki and the Department of Health and Human Services Belmont Report.	Not Applicable	
Studies involving **human participants:** For publication of **patient photos**, include a statement confirming that consent to publish was obtained.	Not Applicable	
Studies involving experimental **animals**: State details of **authority granting ethics approval** (IRB or equivalent committee(s), provide reference number for approval. Include a statement of compliance with ethical regulations.	Yes	Materials and Methods (Ethical statement and mouse strains)
Studies involving **specimen and field samples:** State if relevant **permits** obtained, provide details of authority approving study; if none were required, explain why.	Yes	Materials and Methods

Dual Use Research of Concern (DURC)	Information included in the manuscript?	In which section is the information available? (Reagents and Tools Table, Materials and Methods, Figures, Data Availability Section)
Could your study fall under dual use research restrictions? Please check biosecurity documents and list of **select agents and toxins** (CDC): https://www.selectagents.gov/sat/list.htm	Not Applicable	
If you used a select agent, is the security level of the lab appropriate and reported in the manuscript?	Not Applicable	
If a study is subject to dual use research of concern regulations, is the name of the **authority granting approval and reference number** for the regulatory approval provided in the manuscript?	Not Applicable	

Reporting

The MDAR framework recommends adoption of discipline-specific guidelines, established and endorsed through community initiatives. Journals have their own policy about requiring specific guidelines and recommendations to complement MDAR.

Adherence to community standards	Information included in the manuscript?	In which section is the information available? (Reagents and Tools Table, Materials and Methods, Figures, Data Availability Section)
State if relevant guidelines or checklists (e.g., **ICMJE, MIBBI, ARRIVE, PRISMA**) have been followed or provided.	Not Applicable	
For **tumor marker prognostic studies,** we recommend that you follow the **REMARK** reporting guidelines (see link list at top right). See author guidelines, under 'Reporting Guidelines'. Please confirm you have followed these guidelines.	Not Applicable	
For **phase II and III randomized controlled trials**, please refer to the **CONSORT** flow diagram (see link list at top right) and submit the CONSORT checklist (see link list at top right) with your submission. See author guidelines, under 'Reporting Guidelines'. Please confirm you have submitted this list.	Not Applicable	

Data Availability

Data availability	Information included in the manuscript?	In which section is the information available? (Reagents and Tools Table, Materials and Methods, Figures, Data Availability Section)
Have **primary datasets** been deposited according to the journal's guidelines (see 'Data Deposition' section) and the respective accession numbers provided in the Data Availability Section?	Yes	Materials and Methods
Were **human clinical and genomic datasets** deposited in a public access-controlled repository in accordance to ethical obligations to the patients and to the applicable consent agreement?	Not Applicable	
Are **computational models** that are central and integral to a study available without restrictions in a machine-readable form? Were the relevant accession numbers or links provided?	Not Applicable	
If publicly available data were reused, provide the respective **data citations in the reference list**.	Not Applicable	
